# Effect of lipo-chitooligosaccharide on early growth of C_4_ grass seedlings

**DOI:** 10.1093/jxb/erv260

**Published:** 2015-06-06

**Authors:** Kiwamu Tanaka, Sung-Hwan Cho, Hyeyoung Lee, An Q. Pham, Josef M. Batek, Shiqi Cui, Jing Qiu, Saad M. Khan, Trupti Joshi, Zhanyuan J. Zhang, Dong Xu, Gary Stacey

**Affiliations:** ^1^Divisions of Plant Science and Biochemistry University of Missouri, Columbia, MO 65211, USA; ^2^Christopher S. Bond Life Sciences Center, University of Missouri, Columbia, MO 65211, USA; ^3^Plant Transformation Core Facility,Division of Biological Sciences, University of Missouri, Columbia, MO 65211, USA; ^4^Department of Statistics, University of Missouri, Columbia, MO 65211, USA; ^5^Informatics Institute, Department of Computer Science, University of Missouri, Columbia, MO 65211, USA

**Keywords:** Lipo-chitooligosaccharide, root growth promotion, non-legume, maize, RNA-seq.

## Abstract

Although lipo-chitooligosaccharides (LCOs) are important signal molecules for plant-symbiont interactions, a number of reports suggest that LCOs can directly impact plant growth and development, separate from any role in plant symbioses. In order to investigate this more closely, maize and *Setaria* seedlings were treated with LCO and their growth was evaluated. The data indicate that LCO treatment significantly enhanced root growth. RNA-seq transcriptomic analysis of LCO-treated maize roots identified a number of genes whose expression was significantly affected by the treatment. Among these genes, some LCO-up-regulated genes are likely involved in root growth promotion. Interestingly, some stress-related genes were down-regulated after LCO treatment, which might indicate reallocation of resources from defense responses to plant growth. The promoter activity of several LCO-up-regulated genes using a β-glucuronidase reporter system was further analysed. The results showed that the promoters were activated by LCO treatment. The data indicate that LCO can directly impact maize root growth and gene expression.

## Highlight

Lipochitooligosaccharide (LCOs) are important molecules for plant-microbe symbiosis but can also serve as plant growth regulators. This study shows that LCO addition induces the expression of genes involved in root growth promotion in C4 grasses.

## Introduction

Plants interact with various beneficial, symbiotic microorganisms such as rhizobial bacteria and mycorrhizal fungi, which provide plants with nutrients. Lipo-chitooligosaccharides (LCOs) are the key signal molecules secreted by these microorganisms to initiate plant symbiotic interactions with plants. LCOs secreted by symbiotic bacteria, rhizobia, are essential for the establishment of the nitrogen-fixing symbiosis between rhizobia and roots of legume plants (Oldroyd and Downie, 2008). Since this symbiosis results in the formation of root nodules, these LCOs are commonly referred to as Nod factors. Recently, LCOs were also identified as the Myc factors produced by arbuscular mycorrhizal fungi, which colonize the roots of most plants to improve plant nutrition and water uptake (Maillet *et al.*, 2011). However, as listed in Table 1, a number of observational reports have suggested a broader role for LCOs as a plant growth regulator impacting plant biomass production, shoot and root growth, lateral root branching, cell cycle, embryogenesis, and seed germination (Khan *et al.*, 2011; Maillet *et al.*, 2011; Prithiviraj *et al.*, 2003; Souleimanov *et al.*, 2002; Sun *et al.*, 2015). In addition, LCO treatment of plants was shown to protect against the detrimental effects of abiotic and biotic stresses (Atti *et al.*, 2005; Duzan *et al.*, 2005; Miransari *et al.*, 2006). Since not all of these studies used the same LCO molecule, it is not clear whether specific LCO chemistry is required. Moreover, there is no available evidence as to the molecular mechanisms by which LCO induces these effects on plant growth and development.

**Table 1.** Summary of publications demonstrating the effects of LCOs on plant growth, development, and stress responses

Promoting effect	Plant species	Nod factor	Reference
Cell division	Tobacco	LCO-IV(18:1)	Rohrig *et al.*, 1995
Embryogenesis	Carrot	NodRlv-V (Ac, 18:4)	De Jong *et al.*, 1993
	Norway spruce	NodNGR*	Dyachok *et al.*, 2002
Germination	Beet, Common, Bean, Upland, Cotton, *Arabidopsis*, Maize, Soybean	LCOBj-V (C18:1, MeFuc) NodBj-V (C18:1, MeFuc) LCO-V (C16:0, MeFuc) LCO-IV (C18:1, Fuc,S-Gro) Chitin pentamer	Prithiviraj *et al.*, 2003
	Barley	NodBj-V (C18:1, MeFuc)	Miransari and Smith, 2009
	Pea, Vetch	NodRlv*	Kidaj *et al.*, 2012
	Canola	NodBj*	Schwinghamer *et al.*, 2015
Photosynthesis	Maize, Soybean	Chitosan pentamer Chitin pentamer	Khan *et al.*, 2002
	Soybean	NodBj-V (C18:1, MeFuc)	Jose Almaraz *et al.*, 2007
	Maize, Soybean	NodBj-V (C18:1, MeFuc)	Khan *et al.*, 2008
Root branching	*Medicago truncatula*	LCO-IV (C16:0,S or C18:1,S) LCO-IV (C16:0 or C18:1)	Maillet *et al.*, 2011 Sun *et al.*, 2015
	Rice	LCO-IV (C16:0,S or C18:1,S) LCO-IV (C16:0 or C18:1) Chitin tetramer	Sun *et al.*, 2015
	*Medicago truncatula*	NodSm-IV (C16:2, S)	Olah *et al.*, 2005
Seedling growth	Maize, Rice, Soybean, *Arabidopsis*	NodBj-V (C18:1, MeFuc) NodBj-V (C18:1, MeFuc) LCO-V (C16:1, MeFuc) LCO-V (transC18:1, MeFuc) LCO-IV (transC18:1, Fuc,S-Gro)	Prithiviraj *et al.*, 2003
	*Arabidopsis*	NodBj-V (C18:1, MeFuc) Chitin pentamer	Khan *et al.*, 2011
Shoot and root growths	Maize, Soybean	NodBj-V (C18:1, MeFuc)	Souleimanov *et al.*, 2002
	Pea, Vetch	NodRlv*	Kidaj *et al.*, 2012
	Rice	LCO-IV (C16:0,S or C18:1,S) LCO-IV (C16:0 or C18:1) Chitin tetramer	Sun *et al.*, 2015
	Maize, *Setaria viridis*	LCO-V (C18:1)	This study
Abiotic stress responses	soybean, Maple Glen, Ac Bravour	NodBj*	Miransari *et al.*, 2006
	Soybean	NodBj-V (C18:1, MeFuc)	Atti *et al.*, 2005
Biotic stress responses	Soybean	NodBj-V (C18:1, MeFuc)	Duzan *et al.*, 2005

*Detailed structure of LCO was not defined in the literature

LCO can induce gene expression in non-legume plants. For example, LCO addition to rice plants induced the expression of a *β-glucuronidase* (*GUS*) reporter gene driven by the *pMsENOD12* promoter derived from the alfalfa *ENOD12* gene, an early inducible gene during the rhizobial nodulation process (Reddy *et al.*, 1998). Similar results were found for the induction of a *pOsENOD40::GUS* fusion in rice roots in response to rhizobial LCO application (Kouchi *et al.*, 1999). These data suggest that a signal transduction pathway, perhaps comparable to that existing in symbiotic legume plants, exists in non-legumes. An obvious explanation is that many non-legumes are infected by mycorrhizal fungi and, hence, this LCO signalling pathway exists to respond to fungal LCO. Indeed, it is now well established that core components of the signal transduction pathway coupling LCO recognition to nodule development or to mycorrhizal arbuscular development are shared, reflecting a common evolutionary origin for these symbioses (Delaux *et al.*, 2014; Venkateshwaran *et al.*, 2013). Recently, it was also shown that *Arabidopsis thaliana*, which is neither nodulation nor mycorrhizal competent, also recognizes LCO resulting in significant changes in innate immunity (Liang *et al.*, 2013). Therefore, there exists the possibility that LCOs are fundamental signalling molecules in plants, playing a distinct and additional role to that shown in plant-symbiont interactions.

The effects of LCO addition on seedling growth of maize (*Zea mays*), a major non-legume, cereal crop were examined. The results showed that LCO treatment did increase root growth. Whole-transcriptome analysis with RNA-seq showed that LCO addition had a significant impact on plant gene expression. Some genes predicted to be involved in root growth promotion were among those significantly regulated by LCO treatment. Promoters of those genes regulated by LCO were isolated and fused to the GUS reporter gene and used to construct stably transformed maize plants. In this way, the effects of LCO were studied in more detail. The data suggest that LCO promotes maize root growth by regulation of specific gene expression, clearly expanding the function of this molecule beyond a symbiotic role.

## Materials and methods

### Lipo-chitooligosaccharide

Lipo-chitooligosaccharide mainly used in this study was LCO-V (C18:1), provided by Novozymes (Franklinton, NC). To prepare a stock solution, the LCO was dissolved in 50% (v/v) of acetonitrile. The final concentration of the solvent under assay conditions did not exceed 0.005% (v/v).

### Plant growth conditions

Maize seeds (*Zea mays* L. cv. B73) were sterilized for 45min with 1% (w/v) sodium hypochlorite solution containing 0.1% (v/v) Triton X-100, and then incubated overnight in a fungicide solution containing ~0.008% (w/v) myclobutanil (Spectracide). The seeds were further sterilized in 10% (v/v) hydrogen peroxide solution for 10min. This sterilization method was required in order to insure that experiments were conducted under axenic conditions. After rinsing with autoclaved water, the seeds were placed embryo-side up onto wet paper towels in a petri dish, in the dark, for germination. Three day-old seedlings were transferred to 10ml of MM liquid medium (Olah *et al.*, 2005) in a 50ml falcon tube and grown on a tilting shaker (Bellco Glass Inc.) in a growth chamber under darkness at 25°C. After overnight incubation, chemical treatments were performed by replacing with the liquid medium containing the LCO, and plants grown further followed by harvesting for RNA extraction. To measure LCO effects on seedling growth, 3-d-old seedlings were transferred to a sterile cyg™ pouch (Mega International) with 20ml MM liquid medium. The zone of the each emergent radicle was marked when seedlings were transferred to the pouches. The pouch was covered by a paper folder in order to shade the roots. Plants were grown vertically in a growth chamber under a 16-h light/8-h dark at 25°C. Under these growth conditions, the marked, lower zone represents new root growth while the designated, upper zone represents roots already present at the time the treatment was started (as shown in Supplementary Fig. S1).

*Setaria* seeds (*Setaria viridis* L. acc. A10.1) were sterilized for 3min with 6% (v/v) sodium hypochlorite solution containing 0.1% (v/v) Tween 20, and then rinsed with autoclaved water. The sterile seeds were plated embryo-side up onto a solid medium containing modified one-tenth-strength Hoagland’s nutrient solution and phytagel 1% (w/v) in a square plate. The plates were placed in the dark for 3 d and subsequently grown in the light. The 3-d-old seedlings were transferred to glass tubes with the Hoagland medium (without nitrogen) containing LCO, and then incubated at 23°C in the light.

### Quantitative real-time reverse transcription-polymerase chain reaction (qRT-PCR)

Total RNA was extracted from maize root tissues using a TRIzol based method (Invitrogen). qRT-PCR was carried out as previously described (Tanaka *et al.*, 2011). All data were normalized to a reference gene, folylpolyglutamate synthase (*FPGS*; GRMZM2G393334), which is one of the most reliable reference genes for qRT-PCR for maize (Manoli *et al.*, 2012). Using Ct value (cycle threshold), gene expression levels relative to the reference gene *FPGS* were calculated for each sample as $2^{-\Delta \text{Ct}}=\;[2^{-(\text{Ct sample})}]/[2^{-(\text{Ct FPGS})}]$ . The values of three replicates were used in a Student’s t test to calculate probabilities of distinct induction or repression, and the average ratio of these values to control treatment was used as 2^-ΔΔCt^ to determine the fold change in transcript level (i.e. LCO treatment vs. mock treatment). Primers used are listed in Supplementary Table S5.

### RNA-seq analysis and identification of differentially expressed genes

Quantitation and quality control of RNA were performed using the Agilent 2100 Bioanalyzer system (Agilent Technologies). Total RNA from maize roots treated with or without 10nM LCO (two time points, two treatments, and three replications; total of 12 samples) were subjected for library construction with index adapters (Illumina TruSeq indexes) and then sequenced for 100 single-end bases in two flow cells using the Illumina HiSeq 2000 (Illumina Inc.). Initial base calling and quality filtering of the mRNA-Seq reads generated with the Illumina analysis pipeline (fastQ format) was performed using a custom Perl script and the default parameters of the Illumina pipeline (http://www.illumina.com). As a result, >85% of filtered reads were obtained. Trimming of the reads used the FASTA/Q Trimmer command of the FASTX-toolkit available in the FastQC software package (http://www.bioinformatics.babraham.ac.uk/projects/fastqc/). Finally, RNA-seq reads with good quality were aligned to the maize reference genome (http://www.phytozome.net) using Tophat (version 1.4.1; http://tophat.cbcb.umd.edu/). The genome indexes for Tophat were built using the bowtie-build command of the bowtie (version 0.12.7; http://bowtie-bio.sourceforge.net) based on the reference genome file as input. Tophat was then run with default parameters to map the trimmed/filtered-reads from each library to the reference genome. ~90% on average of trimmed/filtered-reads (more than 20 million reads for each sample) were uniquely mapped in the maize genome, which were considered for further analysis (Supplementary Table S6).

Low-count reads were filtered and only genes that were reasonably expressed in at least three samples were kept before a Poisson linear mixed-effects model (Blekhman *et al.*, 2010) was applied to the raw read counts separately for each gene. Reasonably expressed genes refer to genes with at least one count for each million mapped reads in one sample (Robinson *et al.*, 2010). The model was fitted using the software R/lme4 package (2.10.0 version) with the library size as the offset value to make the comparison across different samples comparable. Each generalized Poisson linear mixed model included the time effect, treatment effect, the interaction between the time and treatment effects, and the random biological replicate effect to account for the batch effect since each replicate was produced at a different time period. The likelihood ratio tests were then conducted to identify differentially expressed genes between the treatment and control groups for each of the two time points (i.e. LCO treatment vs. Mock treatment). *P*-values for the likelihood ratio tests were obtained, and an adjusted *P*-value (Storey and Tibshirani, 2003) was then computed to produce lists of differentially expressed genes with estimated FDR of 5%. Among these significantly differentially expressed genes, genes with the fold change above two were further considered.

### DNA constructs

To construct a GUS reporter system, a GUS-tNOS cassette without promoter was PCR-amplified from pCAMBIA1391Z using a primer set (Supplementary Table S5). The PCR product was digested by *Alw26*I, and then ligated with pFGC5941(-) vector, which was digested by *EcoR*I and *Pme*I beforehand (Supplementary Fig. S6). Upstream regions (1500–2000bp) of starting codon of the genes were PCR-amplified using primers containing recognition site for restriction enzyme at each 5′ end (Supplementary Table S5). The PCR products were digested by certain restriction enzymes (Supplementary Table S4) and inserted into the modified pFGC5941-GUS vector.

### Maize transformation

Maize transformation was performed as described previously (Lee and Zhang, 2014). The recombinant pFGC5941 binary vector was prepared by inserting each promoter segments for the maize transformation. The recombinant plasmid in an *Agrobacterium tumefaciens* strain, AGL1, was introduced into the immature embryos of sterile ears. The generated plants were moved to soil and grown in a greenhouse to produce seed.

### Histochemical GUS reporter assay

To detect GUS activity, histochemical staining using 1 mM5-bromo4-chloro-3-indoyl f-D-glucuronide and 20% (v/v) methanol was performed following the methods described previously (Jefferson, 1987).

### Fluorescence-based biochemical GUS reporter assay in maize protoplast

To prepare maize root protoplasts, radicles from 3-d-old seedlings were collected and cut using razor blades into approximately 0.5mm pieces, which were collected in enzyme solution containing 10mM MES (pH 5.7), 2% (w/v) Cellulase Onozuka R-10, 0.04% (w/v) Macerozyme R-10, 1mM CaCl_2_, 0.6M mannitol. After 12h incubation in room temperature, protoplasts were washed with W5 buffer containing 2mM MES (pH 5.7), 154mM NaCl, 125mM CaCl_2_, 5mM KCl. The protoplasts (from 50 radicles) were suspended in 300 µl MCa solution containing 4mM MES (pH 5.7), 15mM CaCl_2_, and 0.4M mannitol, and then incubated with 10–20 µg plasmid DNA at room temperature for 5min. Transformation was performed by mixing the protoplast solution with the same volume of PEG buffer containing 40% PEG-4000, 0.1M CaCl_2_, and 0.2M mannitol. After 25min incubation at room temperature, the transformed protoplasts were washed and suspended in 500 µl of W5 buffer. The suspensions were further incubated over night at 22°C in the dark. The transformed protoplasts were used for the GUS reporter assay. Fifty microliters of the transformed protoplasts were disrupted in extraction buffer containing 50mM sodium phosphate buffer (pH 7.0), 10mM disodium ethylenediaminetetraacetic acid, 0.1% (v/v) Triton X-100, and 0.1% (w/v) sodium lauroylsarcosinate, and 10mM 2-mercaptoethanol and incubated at 37°C with 1mM 4-methylumbelliferyl-β-D-glucuronide for the fluorometric assay. 4-methylumbelliferon fluorescence was measured using an Enspire® Multimode Plate Reader (PerkinElmer).

## Results

### LCO enhances root growth of maize seedlings

It was previously reported that LCO treatment of legumes, such as *Medicago truncatula*, can affect root growth and branching (Maillet *et al.*, 2011; Olah *et al.*, 2005; Sun *et al.*, 2015). Very recently, LCO-induced root growth promotion was observed in the C3, non-legume plant, rice, which was significantly attenuated in a *dmi3* mutant (Sun *et al.*, 2015). These data suggest that LCO action on rice requires at least some of the genes found in the common symbiotic signalling pathway originally defined by legume studies.

**Fig. 1.**
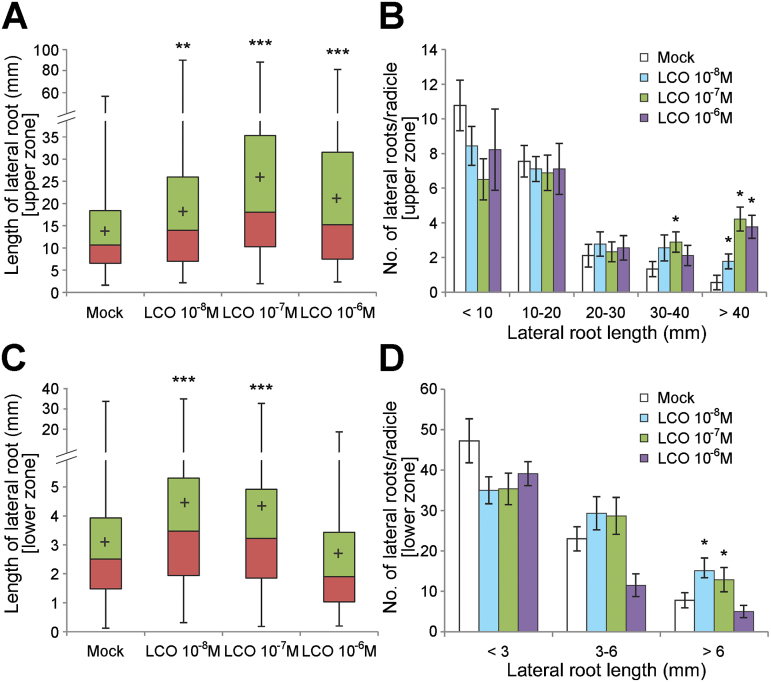
Effects of LCO on lateral root growth of maize seedlings. (A-D) Lateral roots in upper zone in A and B, and lower zone, newly elongated zone in C and D, of radicle were observed separately (see the cartoon in Supplementary Fig. S1 for details). Three-day-old seedlings were grown for 7 d in LCO-containing liquid medium in growth pouches. The box plot in A and C shows with mean value ‘+’ (n = 9), and the median, 25th, and 75th percentiles are marked with line segments across the box. The histogram in B and D shows the distribution of lateral root lengths. Asterisks represent statistically significant differences compared with the control (*P<0.05, **0.001<*P*<0.01, ***P<0.001).

In order to assess whether LCO could also induce root growth on C_4_ grass species, 3-day-old maize seedlings growing hydroponically in plastic growth pouches were used. The levels of LCO used (~10nM) are within the range normally associated with LCO effects during the rhizobial symbiosis (Minami *et al.*, 1996; Walker *et al.*, 2000). Seven days after LCO treatment, the length of the lateral roots of seedlings treated with LCO were significantly longer than that the mock treatment controls (*P* < 0.01; Fig. 1). In contrast, there was no difference in the number of lateral roots. The results suggest that LCO promotes the elongation of lateral roots, but not the formation of new lateral root primordia, including effects on root branching. Radicle growth was also enhanced by LCO treatment although this was not statistically significant (Supplementary Fig. S1). Otherwise, fresh weight, mesocotyl length, and lengths and numbers of nodal roots and seminal roots were measured. However, there were no remarkable effects of LCO treatment on these parameters (Supplementary Fig. S1).

The effects of LCO treatment on the growth of *Setaria viridis* roots were also evaluated. This plant has been proposed as a useful genetic model for studies of C_4_ grass species (Brutnell *et al.*, 2010). Consistent with our findings with maize, the growth of *Setaria* lateral roots was significantly increased (i.e. 1.5-fold longer) by treatment with 10nM LCO in comparison to non-treated control roots (Supplementary Fig. S2). These similar results using at least two distinct C_4_ grass species argue for a general ability of LCO addition to increase lateral root growth.

### Transcriptomic analysis of LCO-treated maize roots

**Fig. 2.**
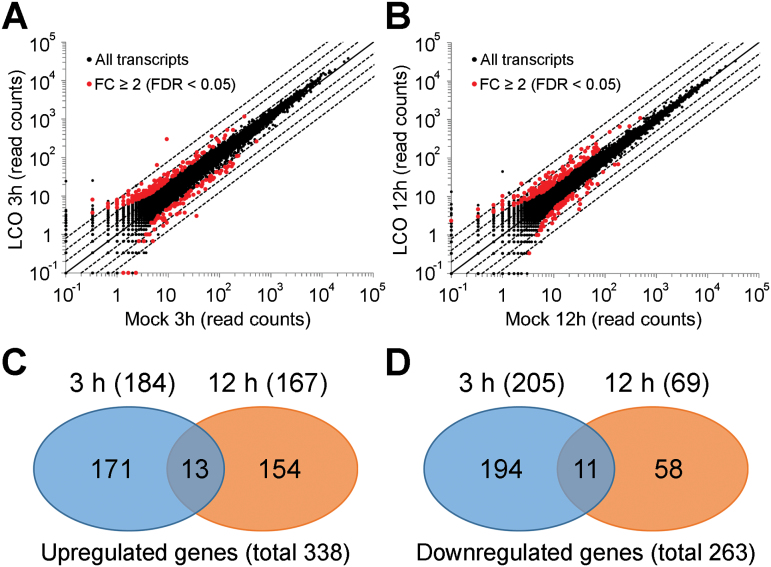
RNA-seq analysis in LCO-treated maize roots. (A, B) Scatter plots showing a comparison of read counts between LCO treatment (*y* axis) and mock control (*x* axis) with logarithmic scales. To allow for log adjustment, genes with 0 read counts were assigned a value of 0.1. Red dots show the genes significantly altered after treatment with 10nM LCO (fold change ≥ 2, FDR < 0.05). Black solid lines indicate the same intensity between two treatments, whereas black dash lines (extended obliquely upward and downward) indicate 2-, 4-, and 8-fold changes, respectively. The data were based on three replicates for each treatment. (C, D) The Venn diagram represents the overlap in differentially expressing genes between different time points. FC; fold change.

In order to further explore how LCO causes growth promotion effects in maize roots, RNA-seq analysis using 3-d-old maize seedlings treated with 10nM LCO for 3h and 12h were performed. Those time points were decided on the basis of previous reports of ENOD gene responses (D’Haeze and Holsters, 2002). As shown in Fig. 2A and 2B, scatter plots of the expression of 63,540 genes were linear in the comparison between LCO treatment and mock treatment, indicating small technical variation. Relative to the mock-treated control plants, a total of 290 genes (184 up; 205 down) and 232 genes (167 up; 69 down) were significantly regulated by the 3h and 12h treatments of LCO, respectively (Fold change ≥ 2) (Fig. 2C, D). A detailed list of the LCO-regulated genes is shown in Supplementary Data 1. Only 13 genes were up-regulated by LCO at 3h and 12h, while 11 genes were down-regulated at both time points (Fig. 2C, D; Supplementary Table S1). The relatively short list of genes responding to LCO treatment may simply reflect a modest physiological response to LCO. However, based on data discussed later in this paper, it more likely reflects the response of only a few specific root cells with the RNA from the non-responding tissue diluting out the measurable response. This would certainly be consistent with the signalling role of LCO in plant symbiotic interactions where only relatively few root cells respond to treatment (Libault *et al.*, 2010). The RNA-seq data were further validated by quantitative real-time reverse transcription-polymerase chain reaction qRT-PCR on genes selected from Supplementary Data 1 (Supplementary Fig. S3). The fold-changes measured by qRT-PCR strongly correlated with those measured by RNA-seq analysis (R square score: 0.66). Of those genes tested by qRT-PCR, 80% were significantly regulated by LCO treatment (Fold change > 2; Student’s t test *P* < 0.05).

### Functional categories of differentially expressed genes

The biological relevance of the LCO-regulated genes was assessed by gene function enrichment analysis using the method available in the web-based tool AgriGo (Du *et al.*, 2010), in which gene ontology terms enriched in each set of the regulated genes were computed by comparing to a default gene reference background with an estimated False Discovery Rate (FDR) value (Supplementary Fig. S4). This method identified different categories between up- and down-regulated genes. Interestingly, genes involved in nutrient reservoir activity were enriched in LCO-down-regulated genes, perhaps indicating reallocation of nutrient resources to plant growth.

In order to further investigate the possible functions of the differentially expressed genes, the LCO-regulated genes were categorized using MapMan ontology terms (Thimm *et al.*, 2004) in combination with the Mercator tool (Lohse *et al.*, 2014) for functional predictions by searching several reference databases (e.g. TAIR, KOG, SwissProt/UniProt, and InterProScans). Although 50% of the LCO-responsive genes still lack a functional annotation, a putative biochemical function could be assigned to the remainder. This analysis implicated a number of the LCO-regulated genes in transcriptional regulation and secondary metabolism (e.g. isoprenoids, phenylpropanoids, and flavonoids) (Supplementary Data 1). In contrast, a significant number of stress-related genes were among those down-regulated by LCO treatment (Supplementary Data 1).

**Fig. 3.**
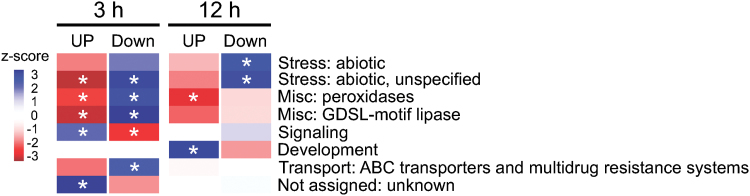
Overrepresented functional categories of genes differentially expressed after LCO treatment. Based on the analysis (see Supplementary Fig. S5 for details), functional categories were identified that showed statistically significant representation (z-score > 1.96 or < -1.96): red to brownish-red, under represented functional category; blue to dark-blue, overrepresented functional category.

Over representation analyses using Fisher’s exact test (Supplementary Fig. S5) showed that signalling and development related genes were overrepresented in the up-regulated gene lists of the 3h and 12h LCO treatments, respectively (Fig. 3; Supplementary Table S2). In contrast, stress-related genes were strongly overrepresented among the LCO-down-regulated genes (Fig. 3). In addition, peroxidases, GDSL lipases, and ABC transporters are also overrepresented in the LCO-down-regulated genes (Fig. 3; Supplementary Table S3), which are also known to function in abiotic and biotic stress responses.

### Comparison of LCO-up-regulated genes with mycorrhiza-induced genes

In order to further identify genes specifically regulated by LCO, the data were compared with previous transcriptome analyses performed using mycorrhizal-infected maize roots (i.e. 9 weeks after inoculation with *Glomus intraradices*) (Willmann *et al.*, 2013). Nineteen and seven genes of our LCO-regulated transcriptome were regulated respectively in mycorrhiza treated roots from control maize and mutant maize (Pi transporter mutant) (Supplementary Fig. S6; Supplementary Data 2), including genes involved in calcium signalling, hormone metabolism, and secondary metabolism, i.e. *calmodulin-binding protein* (*CaMB*), *9-cis-epoxycarotenoid dioxygenase 4* (*NCED4*), and *D-xylulose-5-phosphate synthase* (*DXS*) (Supplementary Data 2). Although there is a time difference between these data and the mycorrhiza-regulated transcriptome, these results are consistent with the notion that mycorrhizal produced LCO could be acting to induce these genes during fungal infection.

### Promoter analysis of LCO-induced genes

**Fig. 4.**
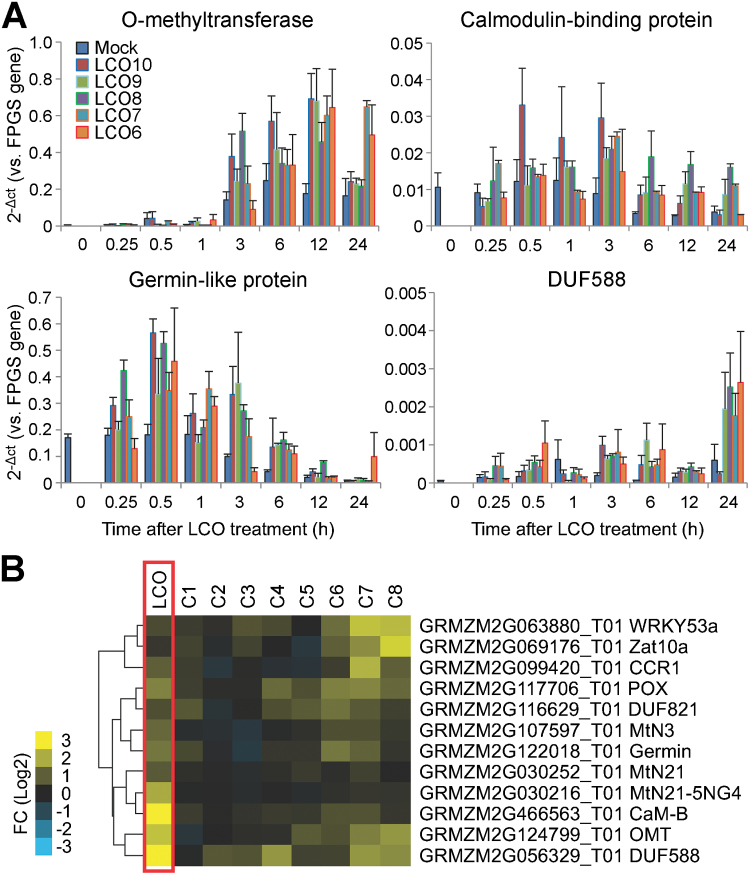
qRT-PCR analysis of LCO-up-regulated genes. (A) Dose- and time-dependent kinetics of LCO-up-regulated genes. LCO10 to LCO6 represent 10^-10^ to 10^–6^ M LCO concentrations. (B) Specificity test of LCO-up-regulated genes. Seedlings were incubated for 3h with LCO or chitin oligomers of increasing length (i.e. N-acetylglucosamine to chitooctaose). WRKY53a and Zat10a, homologs of *Arabidopsis* chitin responsive genes, were used as negative controls (LCO-nonresponsive). C1-C8: chitin monomer to octamer. All chemicals are used at concentration of 10nM. FC: fold change. Data were normalized by the reference gene, *FPGS*, and then converted to 2^-ΔCt^ (A) or Log 2 of 2^-ΔΔCt^ values as fold change, FC (B). See ‘Materials and methods’ in details.

Based on their strong response to LCO treatment, ~20 genes were selected as candidates for promoter cloning upstream of the GUS gene (Supplementary Table S4). The promoter region of each gene was fused with the GUS reporter gene in the modified pFGC5941 vector (Supplementary Fig. S7; Supplementary Table S4). In order to select the best candidates for measurement of the response to LCO treatment, all promoter-GUS constructs were tested by transient transformation in onion epidermal cells (Supplementary Fig. S8). This analysis identified four constructs with detectable GUS expression activity, among which three showed increased GUS staining upon LCO treatment (Supplementary Fig. S8). Other than these four, the others showed no apparent GUS expression in the onion cells, perhaps due to difficulties in cloning a fully functional promoter for these genes. The four active genes were the *CaMB*, *O-methyltransferase family protein* (*OMT*), *germin-like protein* (*GLP*), and *DUF588 domain protein*. Those four genes were further validated by measuring the time- and dose-dependent response, as well as specificity, to LCO treatment by qRT-PCR. The results showed that in these experiments the expression of all four genes strongly responded to LCO (Fig. 4A) and this response was specific to LCO (Fig. 4B).

Transgenic calli expressing the promoter-GUS constructs were generated and used to test the LCO effect on the responses of the four genes mentioned above. As shown in Supplementary Fig. S9, LCO-treated callus stained strongly for GUS in comparison to mock treated control callus (Supplementary Fig. S9A). GUS staining was also performed using roots generated from the transgenic callus (Supplementary Fig. S9B). The results showed that LCO-treated roots exhibited stronger GUS staining in a wider area than non-treated roots.

**Fig. 5.**
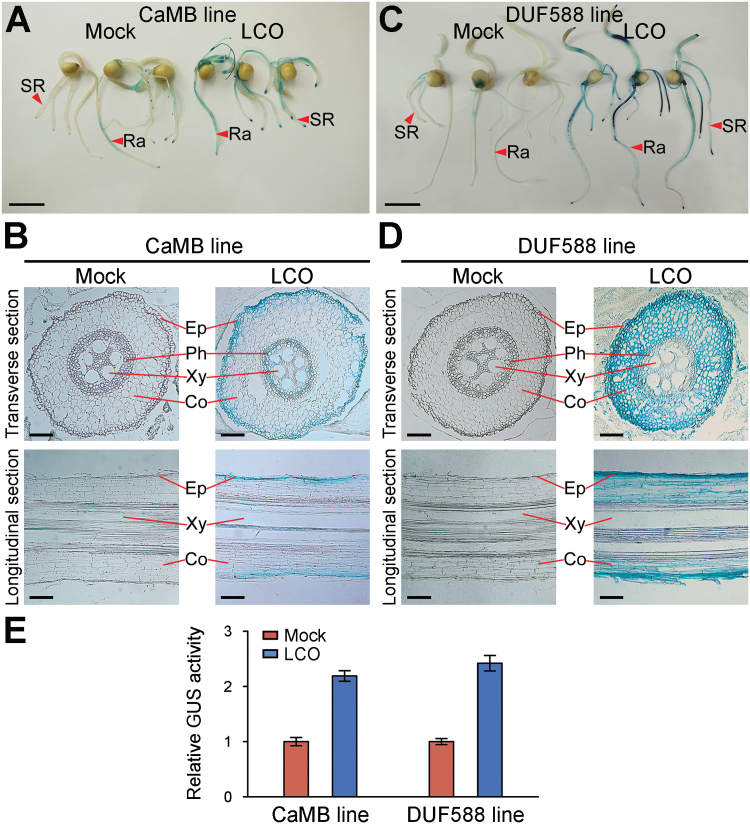
LCO increased promoter activities of LCO-up-regulated genes. (A-D) The expression of p*CaMB*::GUS and p*DUF588*::GUS constructs were shown by histochemical GUS staining in the LCO-treated transgenic maize seedlings in A and C and in transverse and longitudinal seminal root section in B and D. (E) Biochemical GUS assay in the protoplast from the root tissues (n = 5). Co, Cortex; Ep, Epidermis; Ph, Phloem; Ra, Radicle; SR, Seminal root; Xy, Xylem. Scale bars=2cm (A, C), 100 μm (B, D).

In order to support more detailed observations of the LCO effect in maize roots, 16 and 31 independent T1 stable transgenic plants were generated from the transgenic callus expressing the promoter-GUS constructs driven by *CaMB* and *DUF588,* respectively. Three independent transgenic lines for each construct were selected and over eight seedlings of each independent line were tested. This experiment was repeated at least twice. Histochemical GUS assays using the transgenic plants suggested that both *CaMB* (Fig. 5A) and *DUF588* (Fig. 5D) were expressed in root tissues upon LCO addition. Histological sectioning revealed that *CaMB* was moderately expressed in epidermal tissue (Fig. 5B) while *DUF588* was strongly expressed in epidermal and cortical tissues in the LCO-treated roots (Fig. 5E). Fluorescence-based biochemical GUS reporter assays showed that GUS expression was increased up to ~45% in LCO-treated roots in comparison to mock roots (Fig. 5C). These results indicate that *CaMB* and *DUF588* are induced by LCO mainly in the epidermal root layer. This highly localized expression pattern may explain why relatively few LCO-induced genes were identified by the RNA-seq analysis, since the majority of the root tissues did not respond to LCO and mRNA from these tissues would dilute out any signal from those few, LCO responding epidermal cells. Similar results were observed in the transgenic plants expressing the promoter-GUS constructs driven by OMT and GLP (data not shown). Collectively, these results demonstrate that LCO unambiguously activated the promoters selected, suggesting that specific perception of LCOs occurs in maize roots.

## Discussion

### Physiological effect of LCO on maize root growth

LCOs, as Nod factors, are produced by rhizobia and are normally essential for inducing the early steps of legume infection and root nodulation (Maillet *et al.*, 2011). LCOs, as well as short-chain chitooligosaccharides (so-called short chitin), have also been shown to be important early signals during the development of the endomycorrhizal symbiosis (Genre *et al.*, 2013; Maillet *et al.*, 2011). However, are these symbiotic roles the only function of LCO in plants? Published reports suggest that LCOs can have wide ranging effects on both legumes and non-legumes, as well as plants that are not mycorrhizal competent as summarized in Table 1. The current data show that LCO enhances root growth of maize and *Setaria* seedlings (Fig. 1; Supplementary Fig. S2), which may be a general response at least among the grass family.

Recently, Nod factor was suggested to function as a plant growth regulator controlling the balance of other phytohormones by changing the expression of phytohormone-responsive genes including those involved in specific signalling pathways (Breakspear *et al.*, 2014; Souleimanov *et al.*, 2002), suggesting that LCO is not only a symbiotic signal but also a plant growth regulator. Similarly, LCO as Myc factor released by arbuscular mycorrhizal fungi also regulates both plant growth with regulation of phosphate transport related genes, as well as arbuscule formation (Maillet *et al.*, 2011; Tian *et al.*, 2013). Again, the data suggest that LCOs function not only as an important symbiotic signal but also as a regulator of root growth and development to promote further mycorrhizal infection.

### Transcriptional effects of LCO on stress responsive genes

Interestingly, Liang *et al.* (2013) found that *Arabidopsis*, which is neither nodulated nor mycorrhizal competent, also responds to LCO treatment, including regulation of gene expression. Liang *et al.* (2014) suggested that this response to LCOs may have evolved as a way for microbes to specifically suppress plant innate immunity, presumably enhancing colonization and infection. According to this suggestion, this role for LCOs may have predated the role in symbiotic plant interactions as these relationships evolved from a pathogenic to mutualistic lifestyle. If this were, indeed, the case, then plant responses to LCO would be expected to be very widespread with the hallmark that LCO treatment results in a down-regulation of plant stress responses. Since there is a clear negative correlation between activation of plant stress response and plant growth (Lozano-Duran *et al.*, 2013), such a role for LCOs could explain, at least in part, the wide and diverse plant growth promotion properties that have been assigned to these molecules in previous reports (Table 1).

Consistent with this hypothesis, a significant number of stress related genes were among those overrepresented in the list of LCO-down-regulated genes (Fig. 3; Supplementary Table S3), as identified by RNA-seq analysis. Among these were germin-like proteins that have various proposed roles in stress-related processes (Patnaik and Khurana, 2001). In addition, genes encoding peroxidases, GDSL lipases and ABC transporters were among those down-regulated by LCO treatment (Fig. 3; Supplementary Fig. S5). These genes are also known to function in abiotic and biotic stress responses. For example, an *Arabidopsis* GDSL lipase gene was suggested to play an important role in plant immunity for both local and systemic resistance in plants (Kwon *et al.*, 2009). A number of ABC transporters, although encoded by a large gene family, are involved in plant stress responses (Kang *et al.*, 2011).

### Transcriptional effects of LCO on maize root growth

Transcriptomic analysis of maize roots treated with LCO showed that signalling-related genes were overrepresented among the list of genes responding within 3h of LCO treatment. This gene list consisted mostly of S-locus lectin receptor kinases and Ca^2+^ signalling-related proteins (Fig. 3; Supplementary Table S2). An S-locus lectin receptor kinase in soybean was reported to have a role in root growth (Sun *et al.*, 2013). A key early response of legumes to LCO is a marked increase in cytoplasmic calcium levels and, in compatible interactions, the induction of specific calcium spiking (Gough and Cullimore, 2011). Recently, the calmodulin-dependent protein kinase (CCaMK) *OsDMI3*, which acts down-stream of Ca^2+^-spiking, was shown to be involved in root growth mediated by abscisic acid (ABA) signalling (Shi *et al.*, 2014). It was shown that a rice mutant defective in the *OsDMI3* gene exhibited increased root length and was insensitive to ABA (Shi *et al.*, 2014). Notably, comparison of our study with a recent transcriptome study of mycorrhizal-infected maize roots (Willmann *et al.*, 2013) showed that the *CaMB* gene and ABA metabolic genes were up-regulated in both studies (Supplementary Data 2; Supplementary Fig. S6). It is possible that modulation of cellular calcium signalling in maize (e.g. through up-regulation of *CaMB*) by LCO could affect hormonal signalling, including ABA. Moreover, LCO induced the expression of *CaMB*-*GUS* in epidermal tissue and the root tip of maize roots (Fig. 5A, B), consistent with the expression of legume Ca^2+^- or calmodulin-related genes (Tirichine *et al.*, 2006).

A number of LCO regulated genes in maize are annotated as playing a role in secondary metabolism (Supplementary Data 1), most of which are related to isoprenoids, phenylpropanoids, and flavonoids. Flavonoids are synthesized from the phenylpropanoid or acetate-malonate metabolic pathway. Some flavonoids act as cofactors with auxin in root growth and development, although their mode of action remains unclear (Yoshikawa *et al.*, 1986). The maize transcriptome data show that LCO can induce the OMTs (Supplementary Table S1; Supplementary Fig. S9) and predict that OMTs are involved in O-methylation of suberin phenylpropanoid precursors (Held *et al.*, 1993). Suberin is known to be required for root growth and development (Martinka *et al.*, 2012). Alternatively, those OMTs might be involved in lignin biosynthesis together with another LCO-up-regulated gene, cinnamoyl-CoA reductase (Supplementary Data 1). Those two enzymes catalyse key steps in the biosynthesis of monolignols (Tu *et al.*, 2010), although their involvement in the lignin pathway has not yet been established in maize. A recent study of a mutant of an OMT gene in *Medicago* revealed that cell elongation in the root system was strongly regulated through lignin and flavonoid profiles, which may reflect changes in polar auxin transport (Laffont *et al.*, 2010). It is well established in legumes that LCO treatment modulates auxin action, including modification of polar auxin transport (Subramanian *et al.*, 2007). Interestingly, a previous transcriptomic study in *Medicago* reported that both Myc-LCO and Nod-LCO induced specific OMTs, which were among the most strongly responding genes in roots (Czaja *et al.*, 2012). It is intriguing that, in both legumes and non-legumes, LCOs cause the induction of similar genes, some of which are clearly implicated in root growth, including hormonal control. Such data provide useful information to develop specific hypotheses for further experiments.

Isoprenoids, one of the largest groups of secondary metabolites in plants (Withers and Keasling, 2007), are derived from the mevalonate pathway or the methylerythritol phosphate pathway. The RNA-seq data reveal that several isoprenoid metabolic genes encoding DXS and ent-kaurene synthase [involved in gibberellin (GA) biosynthesis pathway], were up-regulated by LCO treatment (Supplementary Data 1). It was also noticed that additional genes implicated in GA biosynthesis and signalling were up-regulated by LCO treatment (Supplementary Data 1) e.g. genes encoding ent-copalyl diphosphate synthase1 (CPS1), carboxyesterase, and iron-dependent oxygenase (GA20OX2), cytochrome P450 / ent-kaurene oxidase (KO1), and GAST protein precursor (GASR2). GAs are key regulators for promoting root growth (Jiang *et al.*, 2007). Moreover, a recent study suggested that Nod factor increased GA levels in *Medicago* roots (Breakspear *et al.*, 2014). Therefore, LCO treatment may affect root elongation in maize seedlings by increased expression of GA biosynthetic genes. It is noteworthy that the metabolic genes for isoprenoids (i.e. DXS) and GA biosynthetic genes were also among those regulated by Myc-LCO and Nod-LCO in *Medicago* (Czaja *et al.*, 2012). MtDXS2 is known to function in late stages of the arbuscular mycorrhizal symbiosis (Floss *et al.*, 2008) but was also suggested to act during presymbiotic stages (Czaja *et al.*, 2012). This gene is also up-regulated by mycorrhizal treatment in maize roots (Willmann *et al.*, 2013).

The expression of several cell wall modifying enzymes were up-regulated by LCO-treatment, including xyloglucan endotransglycosylase, expansins, endo-1,3-beta-glucosidase, UDP-glucose 6-dehydrogenase, polygalacturonase, pectinacetylesterase, etc. (Supplementary Data 1). These genes may also contribute cell growth for maize root elongation (Fig. 1). This transcriptional study has provided insights into the molecular crosstalk that occurs between the LCOs and maize root growth.

### Action of LCO in plant growth regulation

LCOs are remarkable molecules and it is now clear that a wide variety of plants can recognize these molecules and respond in a variety of ways, including suppression of innate immunity (Liang *et al.*, 2013). In contrast, longer-chain chitin (degree of polymerization >6) are best known as strong inducers of plant innate immunity (Shibuya and Minami, 2001; Stacey and Shibuya, 1997; Wan *et al.*, 2004). These contrasting activities are made more interesting by the fact that both LCO and chitin signals are recognized in plants through lysin-motif (LysM) receptors on the plasma membrane (Liang *et al.*, 2014). It is intriguing how such similar molecules, all involving a simple chitin backbone, can elicit such diverse effects in plants, i.e. innate immunity, suppression of innate immunity, symbiotic development, and plant growth promotion, through interaction with similar, evolutionarily related receptors. Moreover, it is now clear that both LCO and chitin signals act on a wide variety of plants, including legumes, non-legumes, dicots, and monocots, regardless of whether they are mycorrhizal or non-mycorrhizal (Liang *et al.*, 2014). Indeed, the ability to recognize chitin appears to be a very ancient trait also found in animals (Liang *et al.*, 2014).

The action of LCOs has been exhaustively studied in relation to the role in legume-rhizobial interactions and to a significant extent in the plant-mycorrhizal symbiosis. It might be useful to have an alternative model system in which LCO action can be studied independent of these symbioses. *Arabidopsis* presents one such system since it is neither nodulated nor mycorrhizal but still responds to LCO (Liang *et al.*, 2013). Maize could perhaps serve as a comparable monocot model. Access to maize transgenic lines generated through this study, which contain promoter-GUS fusions that specifically respond to LCO action, will provide a useful tool to further such investigations. Along with these insights, this study helps explain the function of LCO in maize and provides a critical clue for future genetic studies on host and symbiont interactions in plants.

## Supplementary data

Supplementary data are available at *JXB* online.

Supplementary Data S1. List of LCO-regulated genes.

Supplementary Data S2. Comparison of transcriptome of LCO-regulated transcriptomes in this study with the maize response to mycorrhizal infection as reported by Willmann *et al.* (2013).

Supplementary Fig. S1. Effects of LCO on early growth of maize seedlings.

Supplementary Fig. S2. LCO promotes lateral root growth on model C_4_ grass *Setaria* seedlings.

Supplementary Fig. S3. Validation of RNA-seq data by qRT-PCR.

Supplementary Fig. S4. Cross comparison of enrichment analysis results between LCO-induced up- and down-regulated genes at 3h and 12h.

Supplementary Fig. S5. Over representation analysis of genes differentially expressed after LCO treatment.

Supplementary Fig. S6. Comparison of the transcriptional response of LCO-treated maize roots with previously published results from mycorrhizal infected roots.

Supplementary Fig. S7. Plasmid map of the pFGC5941-GUS vector used in the present study.

Supplementary Fig. S8. Promoter activity test after biolistic bombardment transformation.

Supplementary Fig. S9. LCO increases promoter activity of LCO-up-regulated genes.

Supplementary Table S1. Genes regulated by LCO at both time points, 3h and 12h.

Supplementary Table S2. Overrepresented genes in the pool of LCO-up-regulated genes.

Supplementary Table S3. Overrepresented genes in the pool of LCO-down-regulated genes.

Supplementary Table S4. List of candidate genes for promoter-GUS construct.

Supplementary Table S5. Primers used in this study.

Supplementary Table S6. Quality control and mapping results of RNA-seq data.

Supplementary Data
