# Supplementary material for: Effect of lipo-chitooligosaccharide on early growth of C4 grass seedlings
Source: J Exp Bot. 2015 Jun 6;66(19):5727–38. doi: 10.1093/jxb/erv260 (PMC4566972; doi:10.1093/jxb/erv260)
Supplement: Supplementary Data [file supp_66_19_5727__index.html]

Effect of lipo-chitooligosaccharide on early growth of C4 grass seedlings — Effect of lipo-chitooligosaccharide on early growth of C4 grass seedlings — Supplementary Data 

# Effect of lipo-chitooligosaccharide on early growth of C4 grass seedlings

## Supplementary Data

Data files

- Supplementary Data - Supplementary Data
- Supplementary Data - Supplementary Data
- Supplementary Data - Supplementary Data
