# Supplementary material for: Effect of lipo-chitooligosaccharide on early growth of C4 grass seedlings
Source: J Exp Bot. 2015 Jun 6;66(19):5727–38. doi: 10.1093/jxb/erv260 (PMC4566972; doi:10.1093/jxb/erv260)
Supplement: Supplementary Data [file supp_erv260_jexbot148957_file001.pdf]

Supplementary Information (SI) appendix

Effect of lipo-chitooligosaccharide on early growth of C4 grass seedlings

Kiwamu Tanaka[†], Sung-Hwan Cho[†], Hyeyoung Lee, An Q. Pham, Josef M. Batek, Shiqi Cui, Jing Qiu, Saad M. Khan, Trupti Joshi, Zhanyuan J. Zhang, Dong Xu, Gary Stacey[*]

[†]K.T. and S.-H.C. contributed equally to this work.

[*]Author for correspondence: Gary Stacey (Email: staceyg@missouri.edu)

Supplementary information (SI) contains:

- SI Figures 1-9
- SI Tables 1-6
- SI References

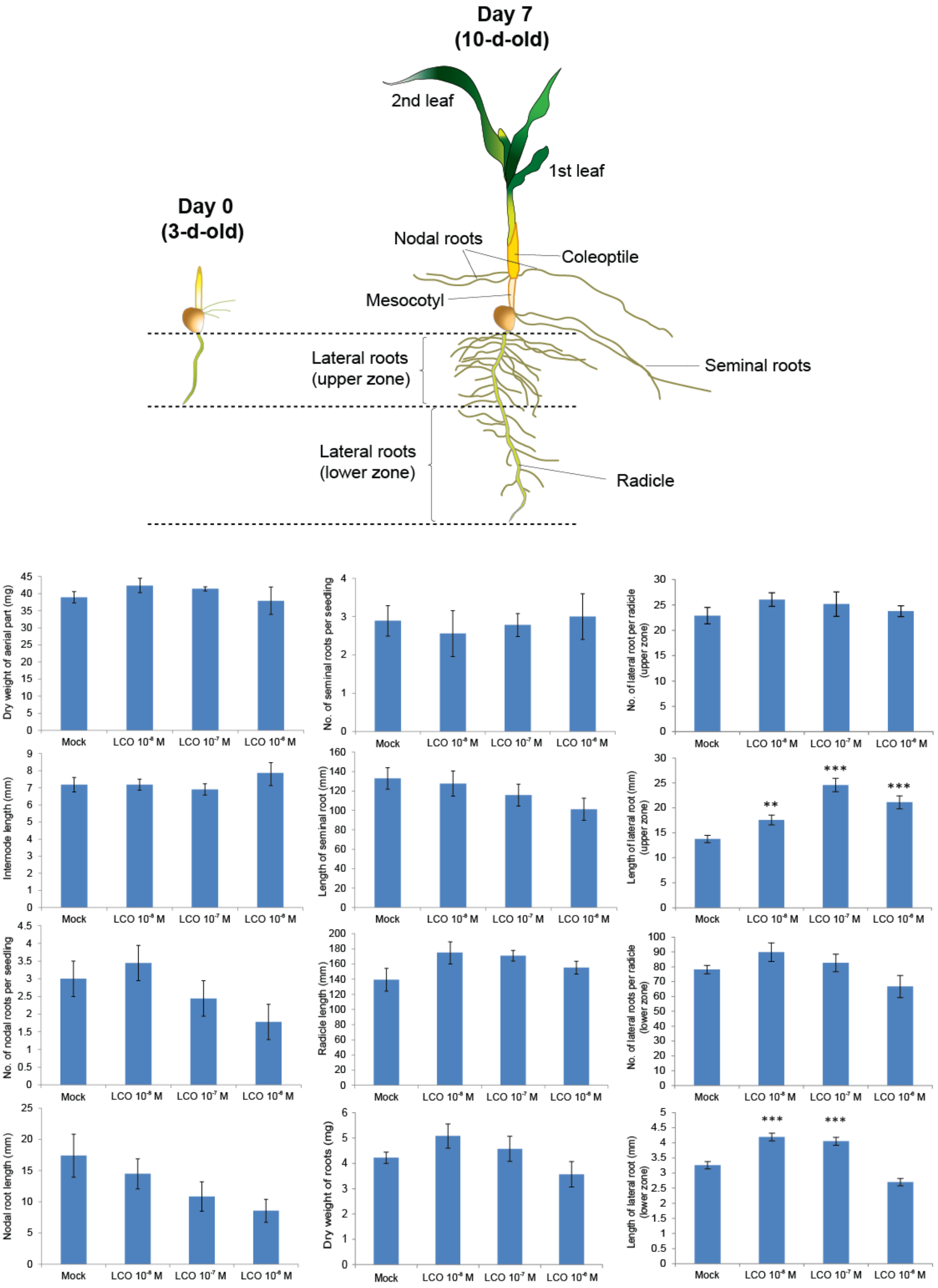

Supplementary Fig. 1. Effects of LCO on early growth of maize seedlings.

Cartoon showing the morphological structures of the maize seedlings at the starting point (day 0) and the ending point (day 7) of hydroponic growth. Histograms show results from a morphometric analysis of the various parts of the maize root after growth in the LCO-containing medium (10^{-8} to 10^{-6} M). Note that there were no remarkable effects of LCO

treatment on all parameters measured except lateral root growth. Asterisks represent statistically significant difference compared with the control (**$0.001 < P < 0.01$, ***$P < 0.001$).

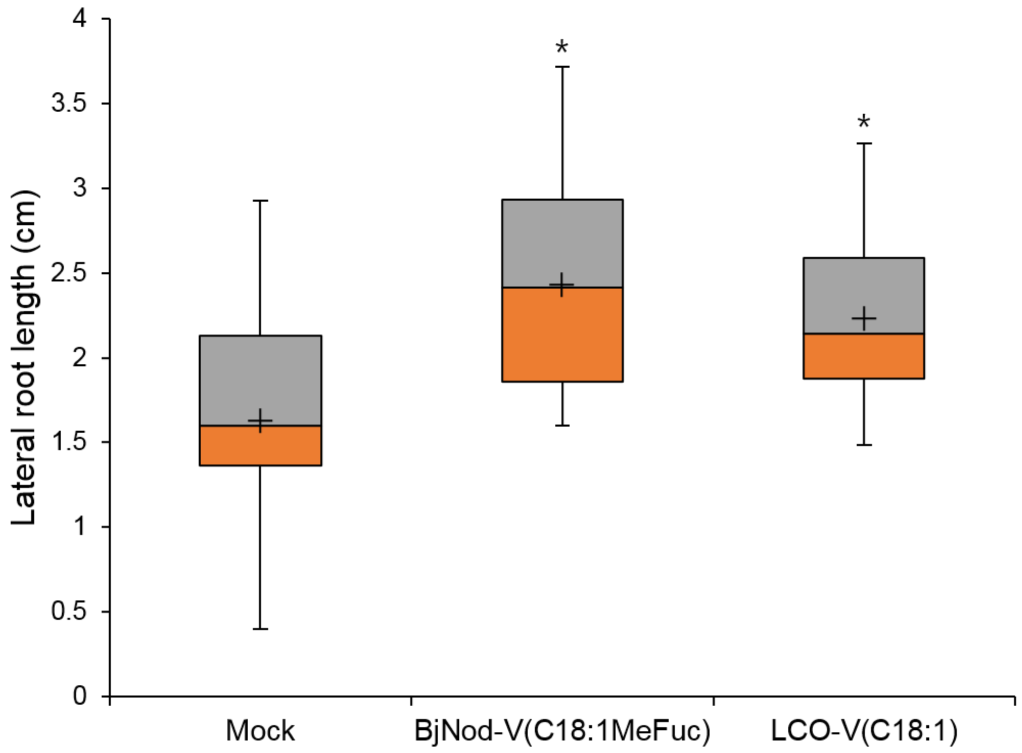

Supplementary Fig. 2. LCO promotes lateral root growth on model C4 grass *Setaria* seedlings. Two day-old seedlings of *Setaria viridis* (A10-1) were transferred into a Hoagland hydroponic medium without nitrogen source and then grown for 10 days with LCOs, BjNod-V (C18:1, MeFuc) and LCO-V (C18:1). Mean value was indicated as "+" on boxplot (n = 10). An asterisk represents a statistically significant difference compared with the control ($P<0.05$).

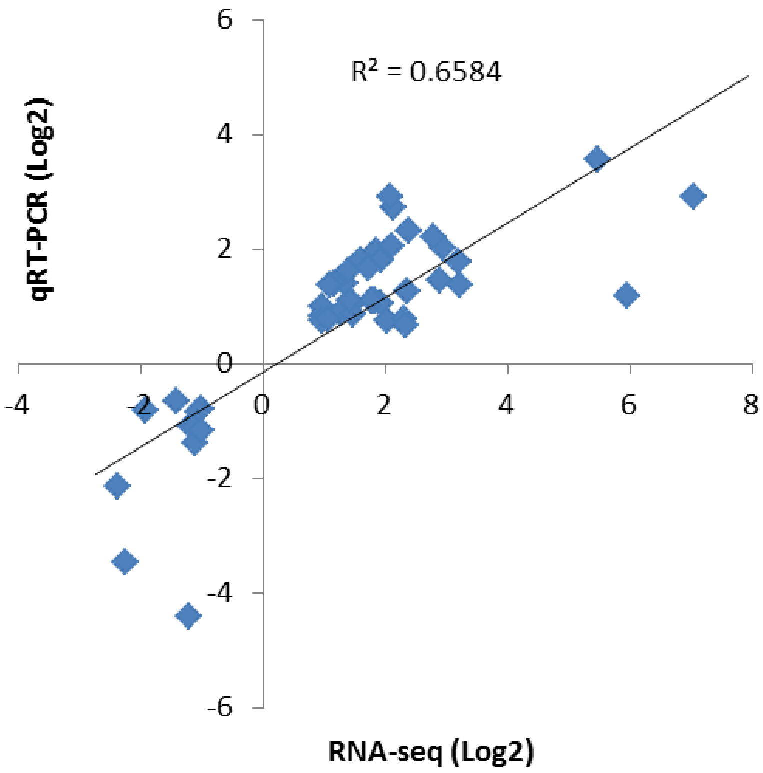

Supplementary Fig. 3. Validation of RNA-seq data by qRT-PCR.

A total of 40 randomly selected genes were validated by qRT-PCR. Graph shows correlation of RNA-seq data and qRT-PCR data on a log scale. Data are the average from three biological replicates. R, Pearson correlation coefficient.

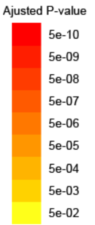

GO Information			Color code				Upregulated at 3 h		Downregulated at 3 h		Upregulated at 12 h		Downregulated at 12 h	
GO Term	Onto	Description	1	2	3	4	Ajusted Pvalue	Num	Ajusted Pvalue	Num	Ajusted Pvalue	Num	Ajusted Pvalue	Num
GO:0005199	F	structural constituent of cell wall					0.017	5	---	---	---	---	---	---
GO:0005102	F	receptor binding					0.017	5	---	---	---	---	---	---
GO:0005576	C	extracellular region					0.0074	8	4.6e-13	20	---	---	1.5e-05	7
GO:0042221	P	response to chemical stimulus					---	---	8.6e-05	13	---	---	---	---
GO:0055114	P	oxidation reduction					---	---	8.8e-05	27	---	---	---	---
GO:0006979	P	response to oxidative stress					---	---	0.00016	9	---	---	---	---
GO:0006812	P	cation transport					---	---	0.02	11	---	---	---	---
GO:0030001	P	metal ion transport					---	---	0.02	9	---	---	---	---
GO:0030145	F	manganese ion binding					---	---	9.4e-15	13	---	---	3.6e-10	7
GO:0045735	F	nutrient reservoir activity					---	---	1.5e-12	13	---	---	4.6e-09	7
GO:0020037	F	heme binding					---	---	2.8e-11	22	0.026	8	---	---
GO:0046906	F	tetrapyrrole binding					---	---	2.8e-11	22	0.026	8	---	---
GO:0005506	F	iron ion binding					---	---	2.8e-11	25	0.024	10	---	---
GO:0046914	F	transition metal ion binding					---	---	1.4e-06	44	---	---	---	---
GO:0043169	F	cation binding					---	---	4.5e-06	48	---	---	---	---
GO:0046872	F	metal ion binding					---	---	4.5e-06	48	---	---	---	---
GO:0043167	F	ion binding					---	---	4.5e-06	48	---	---	---	---
GO:0016491	F	oxidoreductase activity					---	---	4.6e-05	29	---	---	---	---
GO:0016684	F	oxidoreductase activity, acting on peroxide as acceptor					---	---	5.2e-05	9	---	---	---	---
GO:0004601	F	peroxidase activity					---	---	5.2e-05	9	---	---	---	---
GO:0016209	F	antioxidant activity					---	---	0.00013	9	---	---	---	---
GO:0004497	F	monooxygenase activity					---	---	0.00043	10	0.0083	8	---	---
GO:0009055	F	electron carrier activity					---	---	0.0013	12	0.029	8	---	---
GO:0016788	F	hydrolase activity, acting on ester bonds					---	---	0.013	13	---	---	---	---
GO:0048046	C	apoplast					---	---	2.4e-15	14	---	---	1.1e-09	7
GO:0004553	F	hydrolase activity, hydrolyzing O-glycosyl compounds					---	---	---	---	0.024	8	---	---
GO:0003824	F	catalytic activity					---	---	---	---	0.024	56	---	---
GO:0016798	F	hydrolase activity, acting on glycosyl bonds					---	---	---	---	0.024	8	---	---

Supplementary Fig. 4. Cross comparison of enrichment analysis results between LCO-induced up- and down-regulated genes at 3 h and 12 h.

Data represent enrichment categories with adjusted P values and numbers of genes (Num). Color blocks represent the significance level of the enrichment categories. Color code IDs are as follows: 1, upregulated at 3 h; 2, downregulated at 3 h; 3, upregulated at 12 h; and 4, downregulated at 12 h by LCO. Abbreviation: Onto, Ontology categories; P, biological process; C, cellular component; F, molecular function. See http://bioinfo.cau.edu.cn/agriGO/manual.php for further details.

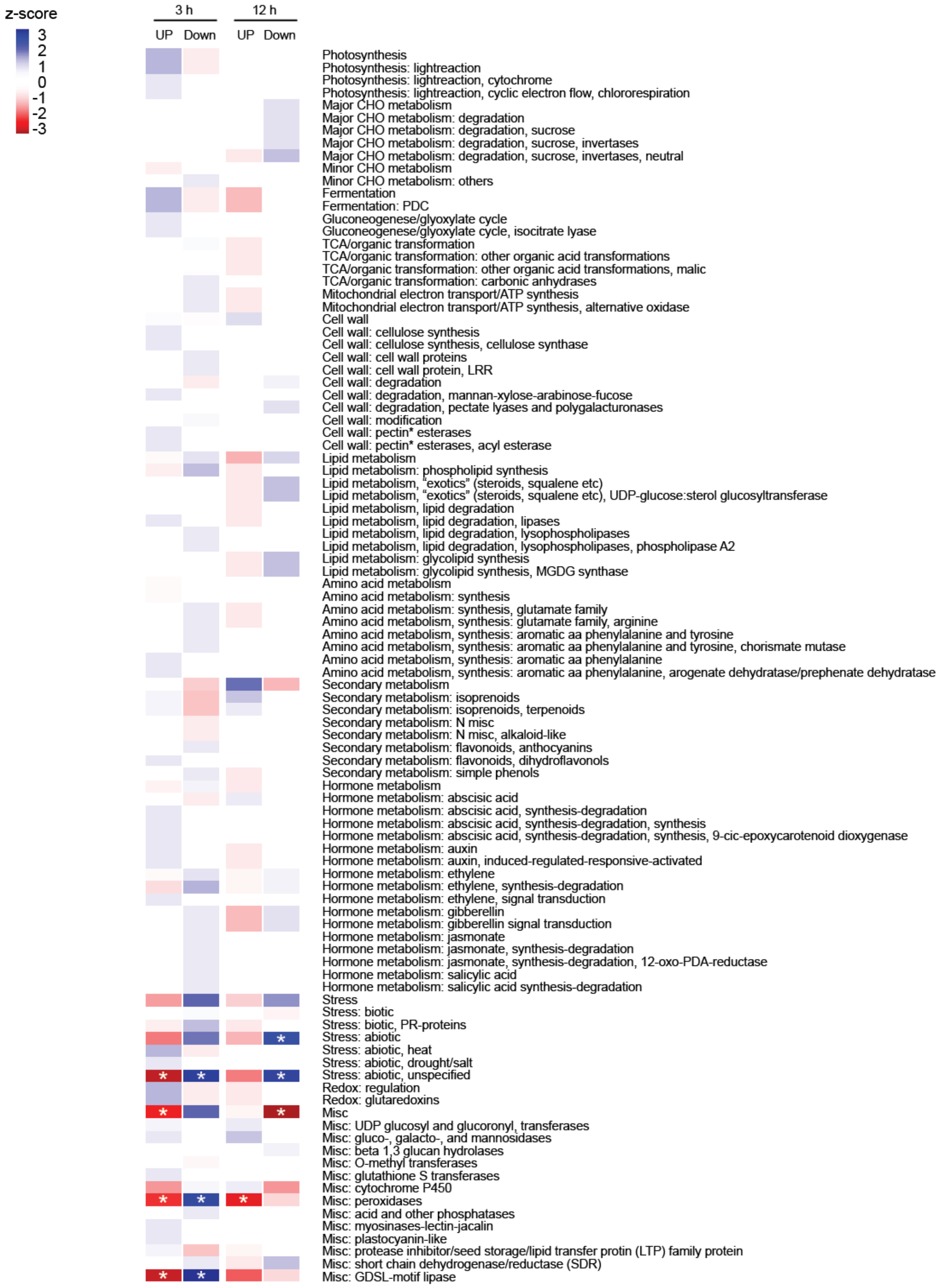
z-score
3
2
1
0
-1
-2
-3
3 h
12 h
UP
Down
UP
Down
Photosynthesis
Photosynthesis: lightreaction
Photosynthesis: lightreaction, cytochrome
Photosynthesis: lightreaction, cyclic electron flow, chlororespiration
Major CHO metabolism
Major CHO metabolism: degradation
Major CHO metabolism: degradation, sucrose
Major CHO metabolism: degradation, sucrose, invertases
Major CHO metabolism: degradation, sucrose, invertases, neutral
Minor CHO metabolism
Minor CHO metabolism: others
Fermentation
Fermentation: PDC
Gluconeogenese/glyoxylate cycle
Gluconeogenese/glyoxylate cycle, isocitrate lyase
TCA/organic transformation
TCA/organic transformation: other organic acid transformations
TCA/organic transformation: other organic acid transformations, malic
TCA/organic transformation: carbonic anhydrases
Mitochondrial electron transport/ATP synthesis
Mitochondrial electron transport/ATP synthesis, alternative oxidase
Cell wall
Cell wall: cellulose synthesis
Cell wall: cellulose synthesis, cellulose synthase
Cell wall: cell wall proteins
Cell wall: cell wall protein, LRR
Cell wall: degradation
Cell wall: degradation, mannan-xylose-arabinose-fucose
Cell wall: degradation, pectate lyases and polygalacturonases
Cell wall: modification
Cell wall: pectin* esterases
Cell wall: pectin* esterases, acyl esterase
Lipid metabolism
Lipid metabolism: phospholipid synthesis
Lipid metabolism, “exotics” (steroids, squalene etc)
Lipid metabolism, “exotics” (steroids, squalene etc), UDP-glucose:sterol glucosyltransferase
Lipid metabolism, lipid degradation
Lipid metabolism, lipid degradation, lipases
Lipid metabolism, lipid degradation, lysophospholipases
Lipid metabolism, lipid degradation, lysophospholipases, phospholipase A2
Lipid metabolism: glycolipid synthesis
Lipid metabolism: glycolipid synthesis, MGDG synthase
Amino acid metabolism
Amino acid metabolism: synthesis
Amino acid metabolism: synthesis, glutamate family
Amino acid metabolism, synthesis: glutamate family, arginine
Amino acid metabolism, synthesis: aromatic aa phenylalanine and tyrosine
Amino acid metabolism, synthesis: aromatic aa phenylalanine and tyrosine, chorismate mutase
Amino acid metabolism, synthesis: aromatic aa phenylalanine
Amino acid metabolism, synthesis: aromatic aa phenylalanine, arogenate dehydratase/prephenate dehydratase
Secondary metabolism
Secondary metabolism: isoprenoids
Secondary metabolism: isoprenoids, terpenoids
Secondary metabolism: N misc
Secondary metabolism: N misc, alkaloid-like
Secondary metabolism: flavonoids, anthocyanins
Secondary metabolism: flavonoids, dihydroflavonols
Secondary metabolism: simple phenols
Hormone metabolism
Hormone metabolism: abscisic acid
Hormone metabolism: abscisic acid, synthesis-degradation
Hormone metabolism: abscisic acid, synthesis-degradation, synthesis
Hormone metabolism: abscisic acid, synthesis-degradation, synthesis, 9-cic-epoxycarotenoid dioxygenase
Hormone metabolism: auxin
Hormone metabolism: auxin, induced-regulated-responsive-activated
Hormone metabolism: ethylene
Hormone metabolism: ethylene, synthesis-degradation
Hormone metabolism: ethylene, signal transduction
Hormone metabolism: gibberellin
Hormone metabolism: gibberellin signal transduction
Hormone metabolism: jasmonate
Hormone metabolism: jasmonate, synthesis-degradation
Hormone metabolism: jasmonate, synthesis-degradation, 12-oxo-PDA-reductase
Hormone metabolism: salicylic acid
Hormone metabolism: salicylic acid synthesis-degradation
Stress
Stress: biotic
Stress: biotic, PR-proteins
Stress: abiotic
Stress: abiotic, heat
Stress: abiotic, drought/salt
Stress: abiotic, unspecified
Redox: regulation
Redox: glutaredoxins
Misc
Misc: UDP glucosyl and glucoronyl, transferases
Misc: gluco-, galacto-, and mannosidases
Misc: beta 1,3 glucan hydrolases
Misc: O-methyl transferases
Misc: glutathione S transferases
Misc: cytochrome P450
Misc: peroxidases
Misc: acid and other phosphatases
Misc: myosinases-lectin-jacalin
Misc: plastocyanin-like
Misc: protease inhibitor/seed storage/lipid transfer protin (LTP) family protein
Misc: short chain dehydrogenase/reductase (SDR)
Misc: GDSL-motif lipase

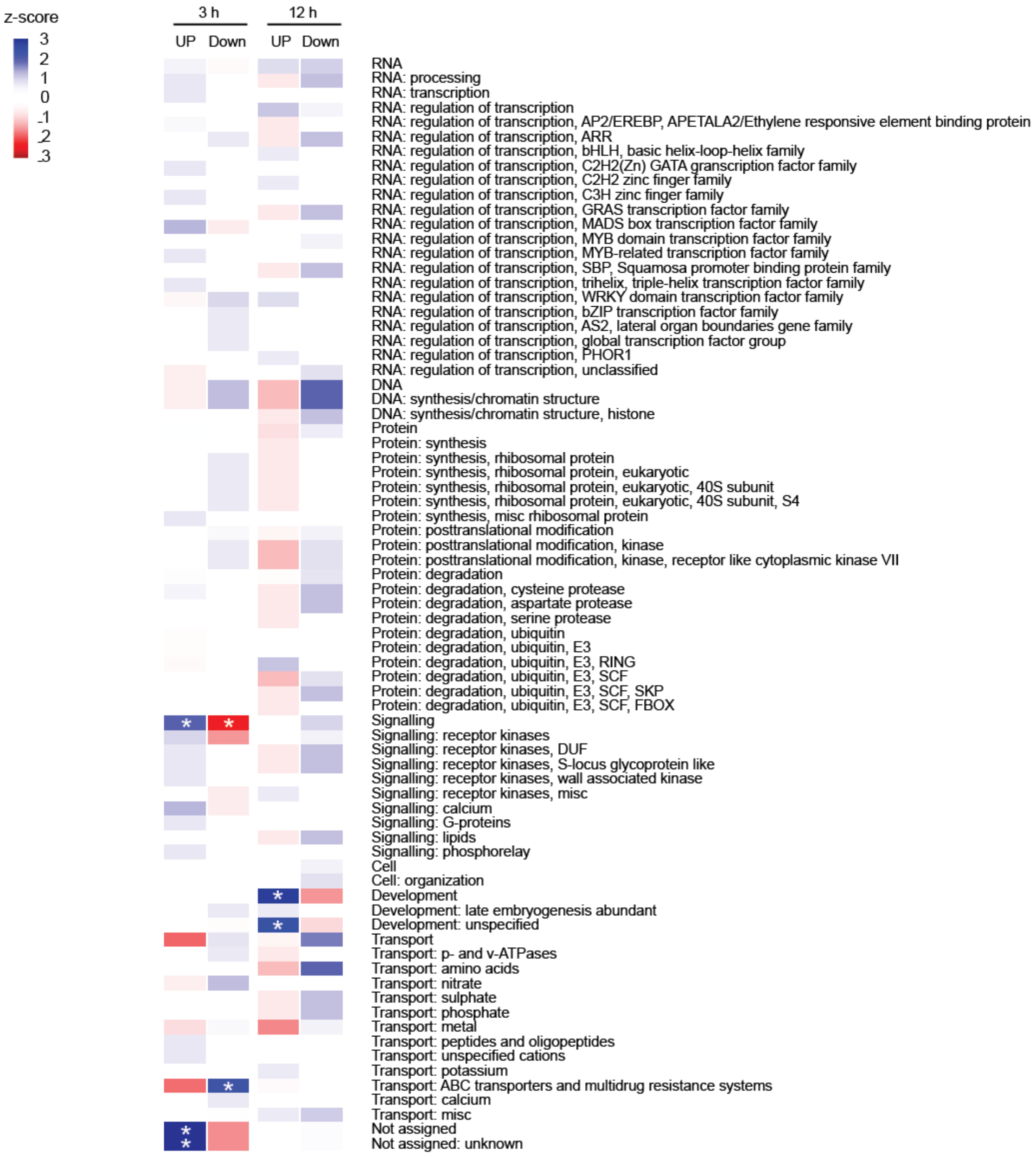

Supplementary Fig. 5. Overrepresentation analysis of genes differentially expressed after LCO treatment.

The analysis of representation of LCO-induced differentially expressed genes within functional categories was performed with the PageMan tool of MapMan software (Thimm *et al.*, 2004). Data points in each category which exceeded the fold change value of 1.0 were tested using Fisher's exact test. Scale bar shows that Z-transformed p-value (z-score). Under-represented functional categories were colored red to brownish-red, while over-represented functional

categories were blue to dark-blue. Asterisks indicate statistically significant representation with z-score > 1.96 (which represents a p-value > 0.05).

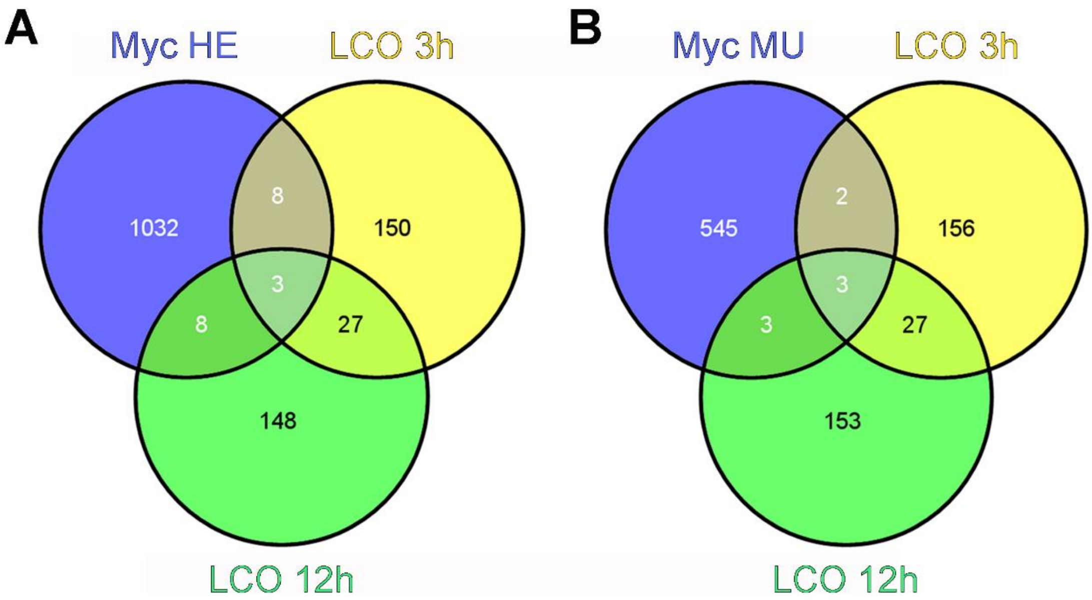

Supplementary Fig. 6. Comparison of the transcriptional response of LCO-treated maize roots with previously published results from mycorrhizal infected roots.

(A, B) Venn diagram displays the overlapped genes between our study and mycorrhizal-treatment study (Willmann *et al*., 2013). Myc HE in A shows mycorrhizal-upregulated genes in maize roots. Myc MU in B shows mycorrhizal-upregulated genes in mutant maize roots, *pht1;6* (Pi transporter mutant). See Supplementary Data 2 for details.

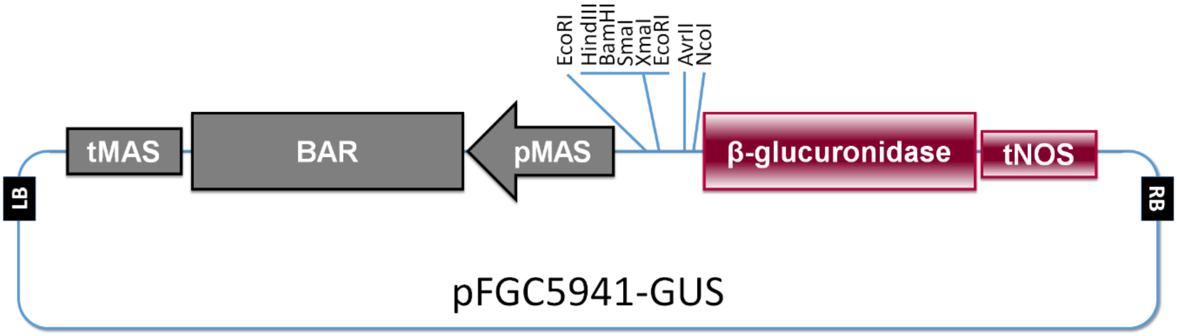

Supplementary Fig. 7. Plasmid map of the pFGC5941-GUS vector used in the present study. The vector was made by replacing a p35S-Ω-tOCS cassette in pFGC5941(-) with a GUS-tNOS cassette from pCAMBIA1391Z (see Materials and Methods for details).

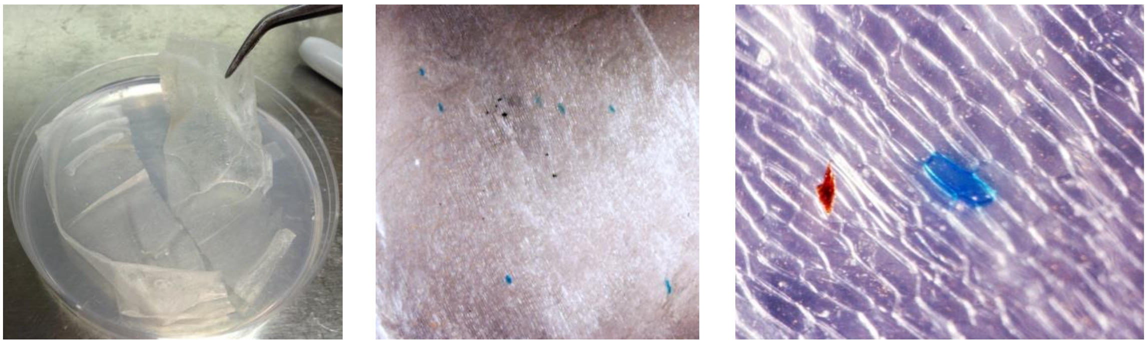

Gene ID	Annotation	Spot counts	
		Mock	LCO
GRMZM2G466563	Calmodulin-binding protein	18	10
GRMZM2G124799	O-methyltransferase family protein (OMT)	10	13
GRMZM2G122018	Germin-like protein | Cupin domain containing protein	12	15
GRMZM2G056329	DUF588 domain containing protein	0	28
GRMZM2G409726	*ZmUbi1*	7	8
X68032	*MtENOD12*	0	3

Supplementary Fig. 8. Promoter activity test after biolistic bombardment transformation. The inner epidermis of the onion bulb's cataphylls (so-called onion peels) was biolistically bombarded and then incubated overnight in the dark on MS medium. The tissues were cut in half (as shown in the left picture) and separately incubated with or without LCO, followed by GUS staining. The picture shows the bombarded tissues with *pZmUbi1::GUS* construct. Table shows the effect of LCO treatment on GUS staining. *MeENOD12* promoter was used for a positive control.

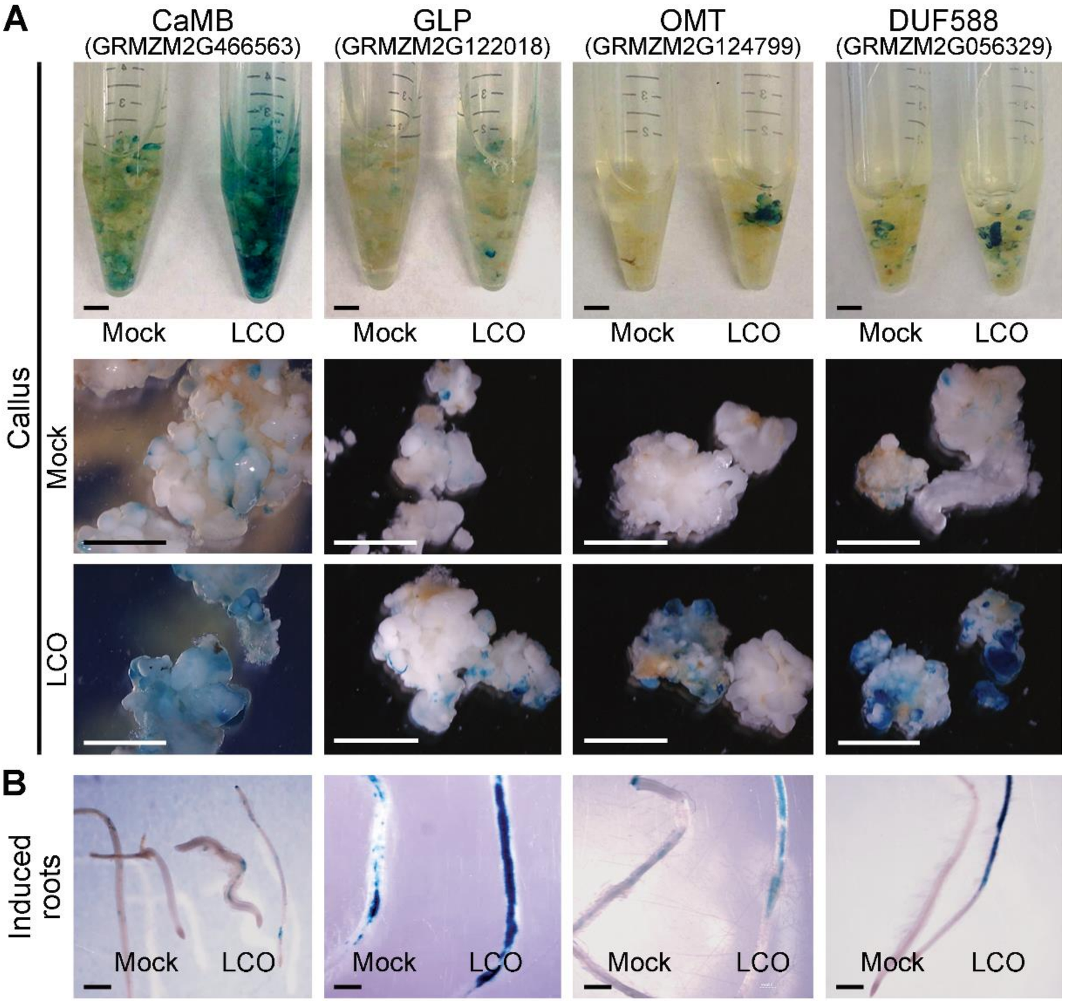

Supplementary Fig. 9. LCO increases promoter activity of LCO-upregulated genes.

(A, B) Transformant calli, induced roots from the calli, and stable transgenic maize were generated (see Materials and Methods for details), which express a *GUS* gene driven by the promoters of the following four genes: CaMB; Calmodulin binding protein; GLP, germin-like protein; OMT, O-methyltransferase; DUF588, domain of unknown function. Tissues were randomly picked and incubated with 10 nM LCO for 4 h, followed by histochemical GUS staining. Scale bars=5 mm (A), 2 mm (B).

Supplementary Table 1. Genes regulated by LCO at both time points, 3 h and 12 h

Gene ID	3 h (LCO vs. Mock)		12 h (LCO vs. Mock)		Annotation by Mapman's functional category		
	Log2 FC	FDR	Log2 FC	FDR	Bin code	Bin name	Description
GRMZM2G016922_T01	1.07	3.2E-04	1.13	5.3E-08	16.1.5	secondary metabolism.isoprenoids.terpenoids	ent-kaurene synthase
GRMZM2G099420_T01	3.26	0.0E+00	2.13	3.7E-14	16.8.3	secondary metabolism.flavonoids.dihydroflavonols	cinnamoyl CoA reductase
GRMZM2G122018_T01	1.24	0.0E+00	1.04	3.8E-08	20.2.99	stress.abiotic.unspecified	Germin-like protein (similar to GLP4)
GRMZM2G124799_T01	1.46	0.0E+00	1.88	0.0E+00	26.6	misc.O-methyl transferases	O-methyltransferase (similar to ZRP4)
GRMZM2G102863_T01	1.63	3.9E-06	2.27	7.8E-08	26.6	misc.O-methyl transferases	O-methyltransferase (similar to ZRP4)
GRMZM2G020761_T01	1.17	1.9E-03	1.03	4.6E-02	26.10	misc.cytochrome P450	Cytochrome P450 (similar to 72A1 and 709B2)
GRMZM2G066978_T01	1.53	8.1E-03	1.37	3.9E-05	26.16	misc.myrosinases-lectin-jacalin	Salt stress-induced mannose-binding lectin
GRMZM2G315431_T01	1.16	4.2E-02	1.35	4.4E-02	29.5.11.4.2	protein.degradation.ubiquitin.E3.RING	U-box domain-containing protein
GRMZM2G466563_T01	1.63	0.0E+00	2.30	9.6E-15	30.3	signalling.calcium	Calmodulin-binding protein
GRMZM2G116629_T01	2.35	3.3E-04	2.05	9.4E-05	34.99	transport.misc	DUF821 (CAP10-like, LPS-modifying protein)
GRMZM2G088121_T01	2.04	5.0E-04	1.71	1.2E-02	34.99	transport.misc	DUF538
GRMZM2G354376_T01	1.74	4.0E-02	1.34	3.1E-03	35.2	not assigned.unknown	Unknown
GRMZM2G317743_T01	1.70	1.4E-05	1.04	2.1E-09	35.2	not assigned.unknown	Unknown
GRMZM2G089856_T01	-1.03	5.8E-14	-1.63	1.6E-11	17.5.1	hormone metabolism.ethylene.synthesis-degradation	1-aminocyclopropane-1-carboxylate oxidase
GRMZM2G170857_T01	-1.08	2.7E-03	-1.83	8.1E-04	20.2.99	stress.abiotic.unspecified	Germin-like protein
GRMZM2G149714_T01	-1.52	7.8E-11	-1.81	0.0E+00	20.2.99	stress.abiotic.unspecified	Germin-like protein
GRMZM2G074443_T01	-1.52	0.0E+00	-1.53	2.5E-06	20.2.99	stress.abiotic.unspecified	Germin-like protein
GRMZM2G012530_T01	-2.48	7.9E-05	-1.24	6.0E-04	20.2.99	stress.abiotic.unspecified	Germin-like protein
GRMZM2G093076_T01	-1.45	0.0E+00	-1.09	4.0E-06	20.2.99	stress.abiotic.unspecified	Germin-like protein
GRMZM2G098474_T01	-1.84	1.7E-06	-1.29	1.1E-02	27.3.5	RNA.regulation of transcription.ARR	Transcription regulator/two-component response regulator
GRMZM2G121333_T01	-1.34	8.5E-14	-1.45	2.6E-11	28.1 | 34.99	DNA.synthesis/chromatin structure | transport.misc	alpha/beta-Hydrolase
GRMZM2G097813_T01	-1.03	9.3E-03	-1.44	8.2E-03	34.99	transport.misc	NB-ARC domain-containing disease resistance protein
GRMZM2G058081_T01	-1.33	8.9E-05	-1.39	6.2E-03	-	Unmapped	Unknown
GRMZM2G030772_T01	-1.35	4.3E-06	-1.16	1.7E-06	-	Unmapped	Unknown

Supplementary Table 2. Overrepresented genes in the pool of LCO-upregulated genes

Gene ID	3 h (LCO vs. Mock)		12 h (LCO vs. Mock)		Annotation by Mapman's functional category		
	Log2 FC	FDR	Log2 FC	FDR	Bin code	Bin name	Description
GRMZM2G152120_T01	0.81	1.3E-01	-1.09	2.2E-03	30.2.17	signalling.receptor kinases.DUF 26	S-locus lectin protein kinase family protein
GRMZM2G465999_T01	1.14	3.7E-02	0.06	6.5E-01	30.2.17	signalling.receptor kinases.DUF 26	S-locus lectin protein kinase family protein
GRMZM2G362303_T01	1.47	6.2E-04	-0.70	1.4E-01	30.2.25	signalling.receptor kinases.wall associated kinase	Wall-associated kinase
GRMZM2G340654_T01	0.88	2.1E-02	1.05	1.5E-02	30.2.99	signalling.receptor kinases.misc	Lectin receptor kinase (similar to S-domain 2.5)
GRMZM2G124890_T01	1.03	6.7E-03	0.17	6.1E-01	30.2.99	signalling.receptor kinases.misc	histidine phosphotransfer kinase (similar to AHP4)
GRMZM2G472311_T01	1.06	2.7E-02	0.21	4.8E-01	30.3	signalling.calcium	Calcium-dependent protein kinase (similar to CPK34)
GRMZM2G466563_T01	1.63	0.0E+00	2.30	9.6E-15	30.3	signalling.calcium	Calmodulin-binding protein
GRMZM2G423315_T01	1.29	2.6E-02	0.60	3.1E-01	30.5	signalling.G-proteins	U3 ribonucleoprotein family protein
GRMZM2G089977_T01	-1.51	3.0E-02	28.99	1.8E-02	33.2	development.late embryogenesis abundant	LEA protein
GRMZM2G167998_T01	0.02	5.4E-01	1.01	2.4E-07	33.2	development.late embryogenesis abundant	LEA protein
GRMZM2G084979_T01	1.35	2.6E-02	1.28	1.3E-01	33.99	development.unspecified	LEA hydroxyproline-rich glycoprotein
GRMZM2G019413_T01	0.07	5.3E-01	1.38	2.0E-02	33.99	development.unspecified	nodulin-like protein
GRMZM2G107597_T01	0.13	4.7E-01	2.27	6.2E-04	33.99	development.unspecified	nodulin MtN3 family protein
GRMZM2G030216_T01	1.23	3.0E-02	2.71	1.2E-01	33.99	development.unspecified	nodulin MtN21 family protein
GRMZM2G068973_T01	0.20	4.6E-01	1.02	1.1E-02	33.99	development.unspecified	NAC domain containing protein
GRMZM2G081930_T01	-0.19	2.0E-01	1.04	1.3E-10	33.99	development.unspecified	NAC domain containing protein
GRMZM2G091490_T01	0.60	8.8E-04	1.06	7.3E-05	33.99	development.unspecified	NAC domain containing protein
GRMZM2G127379_T01	-0.66	8.2E-02	1.48	5.1E-05	33.99	development.unspecified	NAC domain containing protein
GRMZM2G074358_T01	-0.66	1.6E-01	2.14	2.3E-04	33.99	development.unspecified	NAC domain containing protein

Supplementary Table 3. Overrepresented genes in the pool of LCO-downregulated genes

Gene ID	3 h (LCO vs. Mock)		12 h (LCO vs. Mock)		Annotation by Mapman's functional category		
	Log2 FC	FDR	Log2 FC	FDR	Bin code	Bin name	Description
GRMZM2G165839_T01	-1.72	0.0E+00	-0.78	3.8E-02	20.2.99	stress.abiotic.unspecified	Germin-like protein
GRMZM2G170857_T01	-1.08	2.7E-03	-1.83	8.1E-04	20.2.99	stress.abiotic.unspecified	Germin-like protein
GRMZM2G012530_T01	-2.48	7.9E-05	-1.24	6.0E-04	20.2.99	stress.abiotic.unspecified	Germin-like protein
GRMZM2G170829_T01	-1.30	3.4E-03	0.00	6.7E-01	20.2.99	stress.abiotic.unspecified	Germin-like protein
GRMZM2G087111_T01	-0.94	1.1E-02	-1.28	3.1E-07	20.2.99	stress.abiotic.unspecified	Germin-like protein
AC190772.4_FGT011	-1.26	4.3E-02	-0.56	5.3E-01	20.2.99	stress.abiotic.unspecified	Germin-like protein
GRMZM2G105940_T01	-1.75	0.0E+00	-0.88	8.2E-02	20.2.99	stress.abiotic.unspecified	Germin-like protein
GRMZM2G157298_T01	-1.91	6.3E-11	-0.24	5.1E-01	20.2.99	stress.abiotic.unspecified	Germin-like protein
GRMZM2G093622_T01	-1.66	2.7E-04	-0.71	3.5E-01	20.2.99	stress.abiotic.unspecified	Germin-like protein
GRMZM2G149714_T01	-1.52	7.8E-11	-1.81	0.0E+00	20.2.99	stress.abiotic.unspecified	Germin-like protein
GRMZM2G074443_T01	-1.52	0.0E+00	-1.53	2.5E-06	20.2.99	stress.abiotic.unspecified	Germin-like protein
GRMZM2G093606_T01	-1.35	3.5E-02	-0.79	3.0E-01	20.2.99	stress.abiotic.unspecified	Germin-like protein
GRMZM2G093076_T01	-1.45	0.0E+00	-1.09	4.0E-06	20.2.99	stress.abiotic.unspecified	Germin-like protein
GRMZM2G042347_T01	-1.01	0.0E+00	-0.80	0.0E+00	26.12	misc.peroxidases	Peroxidase
GRMZM2G117706_T01	-2.16	0.0E+00	0.96	1.9E-04	26.12	misc.peroxidases	Peroxidase
GRMZM2G138450_T01	-1.07	7.5E-11	-0.32	3.5E-01	26.12	misc.peroxidases	Peroxidase
GRMZM2G015280_T01	-1.20	2.5E-02	-0.19	5.1E-01	26.12	misc.peroxidases	Peroxidase
GRMZM2G076562_T01	-1.14	0.0E+00	0.28	4.3E-04	26.12	misc.peroxidases	Peroxidase
GRMZM2G024234_T01	-1.35	0.0E+00	-0.36	8.4E-07	26.12	misc.peroxidases	Peroxidase
GRMZM2G004984_T01	-1.00	0.0E+00	-0.42	0.0E+00	26.12	misc.peroxidases	Peroxidase
GRMZM2G029144_T01	-1.19	6.7E-10	-0.75	4.5E-02	26.12	misc.peroxidases	Peroxidase
GRMZM2G168073_T01	-1.08	0.0E+00	-0.05	5.3E-01	26.12	misc.peroxidases	Peroxidase
GRMZM2G045215_T01	-1.56	0.0E+00	1.11	3.8E-05	26.28	misc.GDSL-motif lipase	GDSL-motif lipase/hydrolase family protein
GRMZM2G048962_T01	-1.72	0.0E+00	-0.82	1.3E-08	26.28	misc.GDSL-motif lipase	GDSL-motif lipase/hydrolase family protein
GRMZM2G070178_T01	-1.33	0.0E+00	0.44	4.5E-04	26.28	misc.GDSL-motif lipase	GDSL-motif lipase/hydrolase family protein
GRMZM2G176230_T01	-1.65	3.7E-06	-0.60	3.6E-02	26.28	misc.GDSL-motif lipase	GDSL-motif lipase/hydrolase family protein
GRMZM2G063464_T01	-1.74	0.0E+00	-0.50	1.8E-07	26.28	misc.GDSL-motif lipase	GDSL-motif lipase/hydrolase family protein
GRMZM2G465046_T01	-1.19	0.0E+00	0.57	7.6E-05	26.28	misc.GDSL-motif lipase	GDSL-motif lipase/hydrolase family protein
GRMZM2G152962_T01	-2.05	6.9E-15	0.51	1.4E-04	26.28	misc.GDSL-motif lipase	GDSL-motif lipase/hydrolase family protein
GRMZM2G060866_T01	-1.39	0.0E+00	-0.51	5.3E-02	26.28	misc.GDSL-motif lipase	Family II extracellular lipase (similar to EXL1)
GRMZM2G414791_T01	-1.22	0.0E+00	-0.31	8.5E-02	26.28	misc.GDSL-motif lipase	Family II extracellular lipase (similar to EXL3)
GRMZM2G036940_T01	-1.89	0.0E+00	-0.51	2.0E-06	34.16	transport.ABC transporters and MDR systems	ABC-2 type transporter family
GRMZM2G361066_T01	-0.56	2.1E-01	-1.12	4.8E-02	34.16	transport.ABC transporters and MDR systems	ABC-2 type transporter family
GRMZM2G049877_T01	-1.38	4.2E-08	0.21	2.9E-01	34.16	transport.ABC transporters and MDR systems	ABC-2 type transporter family
GRMZM2G054332_T01	-1.21	0.0E+00	0.51	1.8E-09	34.16	transport.ABC transporters and MDR systems	ABC-2 type transporter family
GRMZM2G091478_T01	-1.36	0.0E+00	1.12	0.0E+00	34.16	transport.ABC transporters and MDR systems	ABC-2 type transporter family (similar to ATH6)

Supplementary Table 4. List of candidate genes for promoter-GUS construct

Gene ID	RNA-seq (FC-Log2)		Annotation	Promoter cloning	
	3h	12h		Size (bp)	inserted site
GRMZM2G466563	1.96	2.38	Calmodulin-binding protein	1451	EcoRI-NcoI
GRMZM2G124799	1.88	1.95	O-methyltransferase family protein	2049	EcoRI-AvrII
GRMZM2G122018	1.49	1.09	Germin-like protein | Cupin domain containing protein	2584	EcoRI-AvrII
GRMZM2G056329	0.43	1.22	DUF588 domain containing protein	2525	EcoRI
GRMZM2G172491	1.16	0.80	CYP71A24	1504	EcoRI-AvrII
GRMZM2G030790	0.51	1.71	JA-induced protein	2527	EcoRI-NcoI
GRMZM2G030252	1.45	0.07	nodulin MtN21 | EamA-like transporter	2573	EcoRI-AvrII
GRMZM2G117706	-1.82	1.03	Peroxidase	2620	EcoRI-NcoI
GRMZM2G149273	-0.13	1.11	Peroxidase	1443	EcoRI-AvrII
GRMZM2G156127	1.58	0.03	UDP-glucosyl transferase | cytokinin-O-glucosyltransferase	2514	EcoRI-AvrII
GRMZM2G059562	0.73	1.85	WRKY21	1908	EcoRI-NcoI
GRMZM2G381378	0.47	1.61	WRKY47	2500	EcoRI-AvrII
GRMZM2G116629	2.72	2.10	unknown (DUF821: LPS modifying protein | CAP10-like)	2000	EcoRI-AvrII
CaMV 35S	-	-	-	813	EcoRI-AvrII
pSU	-	-	-	425	EcoRI-AvrII
ZmUbi1	-	-	-	1973	EcoRI-AvrII
MtENOD12	-	-	-	1185	EcoRI-AvrII

*The column shows cloned promoter size and insertion site in pFGC5941-GUS vector. See Supplementary Fig. 7 and Materials and methods for details. We also constructed DNA plasmids that contain the GUS gene driven by the constitutive promoters, super ubiquitin promoter (pSU), CaMV35S promoter (35S), and maize ubiquitin 1 promoter (ZmUbi1).

Supplementary Table 5. Primers used in this study

Experiment	Name	Gene ID	Sequences (5' to 3')	Description
qRT-PCR	DUF588	GRMZM2G056329_T01	TCTTGGCATCAGTAGCAGGA	qRT-PCR forward primer
			GTACATGGCTAAGCACGCTCT	qRT-PCR reverse primer
	OMT	GRMZM2G124799_T01	GTTGTGCCATGTGCTTGCTT	qRT-PCR forward primer
			AGAACAGACAAGAGTACATGCGAT	qRT-PCR reverse primer
	CaM-B	GRMZM2G466563_T01	CCCACCAGCAGATACTCACC	qRT-PCR forward primer
			CACACATCACGGTCCTCACA	qRT-PCR reverse primer
	MtN21-5NG4	GRMZM2G030216_T01	TCACACAAATTAAAGCCGCCAA	qRT-PCR forward primer
			TGGATATGGATGGAGCCCTCT	qRT-PCR reverse primer
	MtN21	GRMZM2G030252_T01	TGGCAAATTCAGGTGAAGCG	qRT-PCR forward primer
			AGGTCGAAGATGAGCATCGC	qRT-PCR reverse primer
	Germin	GRMZM2G122018_T01	CTAGGCTTGATCGATCTCTCGAC	qRT-PCR forward primer
			TGGTGAGCAATGACGCTAGG	qRT-PCR reverse primer
	MtN3	GRMZM2G107597_T01	GCAGCACTAGCCAATGCAAA	qRT-PCR forward primer
			GAGATTACGAGGACGCGGAC	qRT-PCR reverse primer
	DUF821	GRMZM2G116629_T01	GGCCTCGCTCCTCTCCA	qRT-PCR forward primer
			GCCGTCCTTTCTCTGTTGCT	qRT-PCR reverse primer
	POX	GRMZM2G117706_T01	ACAAATAGGCAGCTCGCGTCCAC	qRT-PCR forward primer
			TCGATCAGCTGGCTCTCTCGACC	qRT-PCR reverse primer
	CCR1	GRMZM2G099420_T01	TCACGATGTCGTCCAACTGC	qRT-PCR forward primer
			CACCCACGAGCCGATGAAG	qRT-PCR reverse primer
	Zat10a (chitin responsive gene homolog)	GRMZM2G069176_T01	GACCTCAACCTGATGCCTGT	qRT-PCR forward primer
			ATCCTGCGCTTCTTGATCGG	qRT-PCR reverse primer
	WRKY53a (chitin responsive gene homolog)	GRMZM2G063880_T01	ACAGCTCGGTGTACTGTTCC	qRT-PCR forward primer
			TCAGATAGCCTGGGCACAAC	qRT-PCR reverse primer
	FPGS (Folylpolyglutamate synthase)	GRMZM2G393334_T01	ATCTCGTTGGGGATGTCTTG	qRT-PCR forward primer for a reference gene (Manoli et al., 2012)
			AGCACCGTTCAAATGTCTCC	qRT-PCR reverse primer for a reference gene (Manoli et al., 2012)
	TUB (alpha-tubulin)	X73980	CACTGATGTTGCTGTCCTGC	qRT-PCR forward primer for a reference gene (Manoli et al., 2012)
			CGCTGTTGGTGATTTCGG	qRT-PCR reverse primer for a reference gene (Manoli et al., 2012)
DNA construction	GUS-tNOS (without promoter) from pCambia1391Z		**GTCTC**GAATTCATGACCATGATTACGCCAAGCTTGGCT	Alw26I at 5' end (EcoRI cut edge)
			aaacCCCGATCTAGTAACATAGATGACA	
Promoter cloning	Calmodulin-binding protein	GRMZM2G466563_T01	acaACCTGCacaGAATTCGCTGTACTTTGATGAATAAAGGTTAAGACA	BspMI at 5’ end (EcoRI cut edge)
			acaACCTGCacaCTATGGCCTCAGCTCAGCTGATTCAACGATCGAAGA	BspMI at 5’ end (NcoI cut edge)
	O-methyltransferase family protein	GRMZM2G124799_T01	acaACCTGCacaGAATTCTGATTGCTATAAAATAGAGAAACAACTACC	BspMI at 5’ end (EcoRI cut edge)
			acaACCTGCacaCTATGGGGCGTGGGCAAGGGCTTTTGTTATGCTAAG	BspMI at 5’ end (NcoI cut edge)
	Germin-like protein	GRMZM2G122018_T01	acaaaGAATTCCTCGCGGATCCGCTTACAAAAGGCTTACCA	EcoRI at 5’ end
			acaaaCCTAGGTATTGCTAGCTATTTGCTGTCGAGAGATCG	AvrII at 5’ end
	DUF588 domain containing protein	GRMZM2G056329_T01	acaaaGAATTCTCCGGATAATTCATATTTGAACTGCATGTC	EcoRI at 5’ end
			acaaaGAATTCGGCTAAGCACGCTCTGCTTCTTTCCTGGCT	EcoRI at 5’ end
	CYP71A24	GRMZM2G172491_T01	ACCTGCacaaAATTCTAAATGTTTCGACATCAACAAATATATGG	BspMI at 5’ end (EcoRI cut edge)
			ACCTGCacaaCTAGGCCGTCCTTGCTGATCTTGGTCGGAGACGA	BspMI at 5’ end (AvrII cut edge)
	JA-induced protein	GRMZM2G030790_T01	acaACCTGCacaGAATTCTTTCTTGTCTATTATGATTCATCAGCTAGT	BspMI at 5’ end (EcoRI cut edge)
			acaACCTGCacaCTATGGCTCTCTCTGGATTATCTCTCTGTTTCGGTC	BspMI at 5’ end (NcoI cut edge)
	nodulin MtN21	GRMZM2G030252_T01	acaaaGAATTCCGGTTGAGCCGGGAAAAAATTCCGGTTGAA	EcoRI at 5’ end

		acaaaCCTAGGTGCTAGCTGCTTCGCTTCGCTTCGCTTCAC	AvrII at 5’ end
Peroxidase	GRMZM2G117706_T01	acaACCTGCacaGAATTCTGTAAGCTTGAAACCAAATCATTCATGCAC	BspMI at 5’ end (EcoRI cut edge)
		acaACCTGCacaCTATGGGTCTGTCTAATAACTAAGGGTAACTCGATC	BspMI at 5’ end (NcoI cut edge)
Peroxidase	GRMZM2G149273_T01	acaaaGAATTCTCCAACTCATATTCTCATTTGGCTAAAACT	EcoRI at 5’ end
		acaaaCCTAGGATGGTGAAACTCACCGACGTCTCTCCACTC	AvrII at 5’ end
UDP-glucosyl transferase	GRMZM2G156127_T01	acaaaGAATTCGACGTTGCAACATTAGAAATAGTGTGATAA	EcoRI at 5’ end
		acaaaCCTAGGTGAGGCTGTGTTCGGCTGGTATGGTTTCAA	AvrII at 5’ end
WRKY21	GRMZM2G059562_T01	acaACCTGCacaGAATTCAATAGACTACTATCGCTCTAGGTTCTTCTG	BspMI at 5’ end (EcoRI cut edge)
		acaACCTGCacaCTATGGGGCTGCTGCCGCTCTCGCACTGGTGCTTCT	BspMI at 5’ end (NcoI cut edge)
WRKY47	GRMZM2G381378_T01	acaaaGAATTCATGAAAGGAGGAAGGTGCGATAAAATATAT	EcoRI at 5’ end
		acaaaCCTAGGGCATATGCTCGATCGCTCACTAGCTACCAA	AvrII at 5’ end
CaMV 35S		acaaaGAATTCGCATGCCTGCAGCCCACAGATGGT	p35S promoter from pCam1391Z-35S-GFP vector
		acaaaCCTAGGCGTGTTCTCTCCAAATGAAATGAA	p35S promoter from pCam1391Z-35S-GFP vector
pSU		acaaaGAATTCAAGCTTGATGGGAAAACCCCTCACA	Superubiquitin promoter from pAKK1467B
		acaaaCCTAGGGAATTCCTCCGCCTCCTGGTTAGA	Superubiquitin promoter from pAKK1467B
ZmUbi1		acaaaGAATTCCGGTCGTGCCCCTCTCTAGA	ZmUbi1 DQ141598
		acaaaCCTAGGCTGCAGAAGTAACACCAAACA	ZmUbi1 DQ141598
MtENOD12		TTAGGAATTCATATACATGGGGGAG	MtENOD12 promoter substitution for EcoRI
		GGAAGCCATGGTAAGTAGTAATTTT	MtENOD12 promoter substitution for NcoI

Supplementary Table 6. Quality control and mapping results of RNA-seq data

FastQC Result Chart		Mock 3h			LCO 3 h			Mock 12 h			LCO 12 h		
		Rep 1	Rep 2	Rep 3	Rep 1	Rep 2	Rep 3	Rep 1	Rep 2	Rep 3	Rep 1	Rep 2	Rep 3
Original reads		**28314261**	**39349480**	**29524684**	**37289255**	**42719022**	**43961711**	**34796392**	**35222794**	**33877209**	**38912471**	**35348078**	**35389990**
Filtering	Good	24428509	34122193	25441675	32537583	36667333	37987374	30575479	30781330	29587484	34149737	30452684	30962107
	Bad	3885752	5227287	4083009	4751672	6051689	5974337	4220913	4441464	4289725	4762734	4895394	4427883
	%	**86.28%**	**86.72%**	**86.17%**	**87.26%**	**85.83%**	**86.41%**	**87.87%**	**87.39%**	**87.34%**	**87.76%**	**86.15%**	**87.49%**
Trimming (FastX)	Good	21113137	29505262	21943740	28255086	31674342	32687586	26198337	26064530	24957715	29060299	25810089	26417654
	Bad	3315372	4616931	3497935	4282497	4992991	5299788	4377142	4716800	4629769	5089438	4642595	4544453
	%	**86.43%**	**86.47%**	**86.25%**	**86.84%**	**86.38%**	**86.05%**	**85.68%**	**84.68%**	**84.35%**	**85.09%**	**84.75%**	**85.32%**
Mapping (Tophat)	Mapped	19130016	26738424	19893048	25635988	28466347	29607792	23445973	23297196	22136589	26417495	22718375	23260457
	Unmapped	1983121	2766838	2050692	2619098	3207995	3079794	2752364	2767334	2821126	2642804	3091714	3157197
	%	**90.61%**	**90.62%**	**90.65%**	**90.73%**	**89.87%**	**90.58%**	**89.49%**	**89.38%**	**88.70%**	**90.91%**	**88.02%**	**88.05%**

SI References

Thimm O, Blasing O, Gibon Y, Nagel A, Meyer S, Kruger P, Selbig J, Muller LA, Rhee SY, Stitt M. 2004. MAPMAN: a user-driven tool to display genomics data sets onto diagrams of metabolic pathways and other biological processes. *The Plant Journal* **37**, 914-939.

Willmann M, Gerlach N, Buer B, Polatajko A, Nagy R, Koebke E, Jansa J, Flisch R, Bucher M. 2013. Mycorrhizal phosphate uptake pathway in maize: vital for growth and cob development on nutrient poor agricultural and greenhouse soils. *Frontiers in Plant Science* **4**, 533.
